# Aggressive Glioblastoma Cells Enhance the Migratory Persistence and Velocity of Less Aggressive Cells to Promote Tumor Dissemination

**DOI:** 10.1002/smll.202508142

**Published:** 2026-01-08

**Authors:** Fatima‐ezzahra Ait Mohand, Shaked Yemini, Irina Gorobetz‐Cojocari, Ariel M Rubinstein, Assaf Zemel, Nataly Kravchenko‐Balasha

**Affiliations:** ^1^ The Institute of Biomedical and Oral Research Hebrew University of Jerusalem Jerusalem Israel

## Abstract

Glioblastoma multiforme (GBM) is a highly lethal brain cancer driven by aggressive invasion. Epidermal growth factor receptor (EGFR) is frequently amplified in GBM, with the EGFRvIII mutant enhancing the infiltrative features of EGFRwt‐overexpressing cells. Previously, we identified Src as a key cell‐cell communication mediator between EGFRvIII and EGFRwt cells. However, a quantitative biophysical characterization of how EGFRvIII induces this increased infiltration, specifically detailing altered movement parameters and the underlying mechanisms, remains lacking. Using bulk cell culture and 2‐cell microfluidic chips, we quantitatively analyzed the motility of EGFRwt‐overexpressing and EGFRvIII‐expressing GBM cells. We observed that EGFRvIII cells exhibit higher migration velocity and persistence compared to EGFRwt cells, with a highly persistent subpopulation contributing significantly to these differences. Notably, the co‐culture of EGFRvIII cells enhanced the velocity and migration persistence of EGFRwt‐overexpressing cells, leading to increased spreading. Our findings revealed that Src‐mediated cell‐cell communication from EGFRvIII‐expressing to EGFRwt‐overexpressing cells promotes aggressive GBM spreading by increasing both their velocity and migration persistence at the micro‐environmental level. Inhibiting the Src pathway with dasatinib reversed this pro‐migratory effect, markedly reducing their migration persistence. These insights refine our understanding of GBM infiltration and highlight Src inhibition as a promising strategy in EGFRvIII‐positive tumors.

## Graphical abstract

We identify biophysical motility parameters explaining how less aggressive EGFRwt‐overexpressing Glioblastoma cells become more infiltrative when influenced by aggressive EGFRvIII‐expressing cells. Using bulk assays and microfluidic chips, we show that EGFRvIII cells enhance velocity and persistence in EGFRwt cells via Src‐mediated communication. Dasatinib blocks this effect, revealing mechanisms driving infiltration and a potential therapeutic vulnerability.

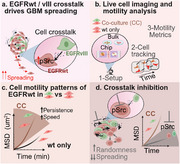

## 1 Introduction

Glioblastoma multiforme (GBM), the most common primary brain malignancy, remains a devastating disease with a median survival of a mere 15 months [1]. It is characterized by an exceptional propensity for infiltration within the brain parenchyma rather than distant extracranial metastases [2]. GBM's invasive growth directly contributes to local tissue damage and tumor recurrence. Despite intensive standard‐of‐care regimens, the majority of patients experience relapse, leading to a dismal overall survival of just 2–9 months [3].

The molecular landscape of recurrent GBM is frequently marked by dysregulation of genes encoding key regulatory proteins, including receptortyrosine kinases (RTKs) [4]. Among these, alterations in the Epidermal Growth Factor Receptor (EGFR) are particularly prevalent. Specifically, EGFR gene amplification, herein referred to as EGFRwt (to distinguish it from other EGFR mutations/variants such as deletions), is observed in approximately 57% of primary GBMs, resulting in high EGFR protein expression. A dominant mutation in GBM is the EGFRvIII variant, which lacks amino acids 6‐273 in its extracellular domain, causing ligand‐independent constitutive receptor activation. Notably, GBM tumors with EGFRwt amplification often harbor a small subpopulation of cells expressing EGFRvIII [5], although EGFRvIII expression is rarely found in the absence of EGFRwt amplification [6]. Accumulating evidence indicates that EGFRvIII‐positive tumors are more malignant than their EGFRvIII‐negative counterparts [7], exhibiting enhanced infiltrative capabilities [8, 9].

In line with these observations, our previous findings revealed that EGFRwt‐expressing cells display enhanced infiltrative properties when cultured in the presence of EGFRvIII‐expressing cells [10]. This increased infiltration was characterized by greater cell‐cell separation distances and higher migration velocities, driven by intercellular communication between the two subpopulations. Specifically, our prior data suggested the involvement of the Src‐mediated signaling pathway in EGFRwt cells, with secreted factors IL‐6 and HGF seemingly amplifying these interactions. This aligns with the well‐established role of Src in promoting cell motility in various cell types, where it influences focal adhesion dynamics, actin cytoskeleton rearrangement, and cell polarity, all of which are crucial for directional cell movement [11, 12]. Notably, this Src activation in EGFRwt cells is specifically mediated by the secreted factors IL‐6 and HGF from EGFRvIII cells, and its specific relevance has been validated by both siRNA‐mediated Src knockdown and in vivo models [10].

Interestingly, GBM cells exhibit a remarkable degree of intrinsic migratory persistence. Single‐cell tracking experiments in isotropic matrigel have identified a subset of GBM cells, termed “highly persistent cells,” that display highly directed trajectories, maintaining their direction of movement for extended periods before reorientation [13]. Similar persistent migration patterns have been observed in GBM cells within brain‐mimicking spheroid models [14]. However, a quantitative biophysical characterization explaining how EGFRvIII cells induce this increased infiltration of EGFRwt cells in EGFRvIII‐positive tumors, specifically determining whether this is driven by the induction of highly persistent migration and describing which movement parameters are altered and the underlying mechanisms, remains lacking.

To address this critical gap in understanding, we sought to quantitatively analyze the motility of GBM cells and explore the specific influence of EGFRvIII signaling on this process. Consequently, we established both bulk cell culture and micropattern chip assays to directly investigate how EGFRvIII‐expressing cells affect the motility of EGFRwt‐expressing cells.

Our findings demonstrate that EGFRvIII cells exhibit both faster and more persistent migration relative to EGFRwt cells. Furthermore, the motility of both cell types displays anisotropy, with migration being preferentially directed along an inherently chosen axis where velocity and persistence are higher. In addition, we identified a subpopulation of highly persistent (HP) cells within both EGFRwt and EGFRvIII populations, which accounted for the most significant differences in motility characteristics between the two cell types. Strikingly, when grown in co‐culture, EGFRvIII cells significantly increased both the velocity and persistence of EGFRwt cells, thereby promoting their dissemination over larger areas; an effect we could counteract with the Src inhibitor dasatinib. These findings collectively suggest that paracrine Src‐dependent signaling from EGFRvIII cells initiates faster and more directed motility of EGFRwt cells, both characterizing and promoting the aggressive infiltration observed in GBM.

## 2 Results

### 2.1 EGFRvIII Cells Migrate Faster and More Persistently along a Preferred Direction than EGFRwt Cells and thus Spread to Larger Areas

EGFRvIII cells have been documented to exhibit more aggressive and malignant behaviors than EGFRwt cells, demonstrating increased spreading in both a 2D scratch assay and a 3D matrigel environment [10]. Two major factors dictate the spreading rate of a cell population: the mean cellular velocity and the persistence time, characterizing the timescale over which a cell moves in a fixed direction. The higher the velocity and the longer the persistence time, the faster a cell population spreads out from an origin. To gain insight into the mechanisms underlying the distinction between EGFRvIII and EGFRwt cells, we sought to quantitatively analyze their motile characteristics. As representatives of the EGFRvIII+ and EGFRwt+ cellular subtypes found in GBM EGFRvIII tumors [10, 15], we used U87EGFRvIII and U87EGFRwt cells [16].

To quantitatively characterize the migratory behavior of the two cell subtypes, we employed time‐lapse microscopy followed by detailed analysis of individual cell trajectories (see Methods and Supporting Information Methods; Figure S1 illustrates the analysis workflow).

**FIGURE 1:**
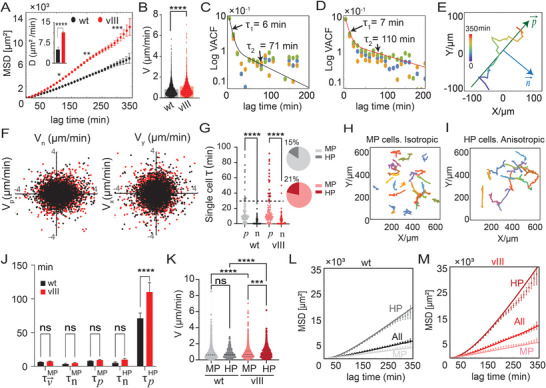
Quantitative analysis of EGFRwt and EGFRvIII subtype motility characteristics: (A) Mean Squared Displacement (MSD) calculated for EGFRvIII cells (vIII, n = 111) and EGFRwt (wt, n= 138) (mean ± SEM, ^*^ *p* < 0.05, ^**^ *p* < 0.01, ^***^ *p* < 0.001, Mann–Whitney U test (two‐tailed)). Inset: Diffusivity (D) coefficient calculated for each culture ((mean ± s.d, ^****^ *p* < 0.0001, Mann–Whitney U test (two‐tailed)). (B) Cell velocity distribution for each group. Bars: Mean ± s.d. (C,D) Velocity autocorrelation function (VACF) for (C) EGFRwt and (D) EGFRvIII cell populations, plotted in log‐linear scale; orange and green dots are the autocorrelation function of the x‐ and y‐components of cell velocity, and blue dots represent the overall VACF. The plots reveal two prominent timescales in the decay of cell velocity correlations. Solid lines are drawn from Equation (4) (methods). (E) A representative cell trajectory showing the calculated trajectory's principal direction, $\vec{p}$, and the normal direction, $\vec{n}$. (F) Scatter plots of: (right panel) the instantaneous velocity components, V_x_ and V_y_, measured along the lab frame coordinate system, and (left panel) the instantaneous velocity components V_p_ and V_n_ along each cell trajectory's principal and normal directions. The data are evaluated for all cells and all trajectory time points; black dots are for the EGFRwt cells and red dots for the EGFRvIII cells (each dot corresponds to a given time point in a given cell trajectory). The elliptical scatter of the data in the V_p_ vs. V_n_ plot reveals the anisotropy of cell movements, with cellular speed being higher along an inherently chosen principal trajectory direction, $\vec{p}$. (G) Distributions of single‐cell persistence‐times along the trajectories' principal and normal directions, $\tau_{p}^{i}$ and $\tau_{n}^{i}$, respectively; black shades are for EGFRwt cells and red shades are for EGFRvIII cells. Each dot represents an individual cell. The dashed line marks a 30‐min cutoff value used to classify the cells into moderately persistent (MP) and highly persistent (HP) cells (^****^ *p* < 0.0001. Kruskal–Wallis test followed by Dunn's multiple comparisons test). Pie charts indicate the percentage of MP and HP cells in the population. (H,I) Representative trajectories of moderately persistent (in (H)) and highly persistent cells (in (I)) from the EGFRwt subpopulation. (J) The extracted persistence times, $\tau_{\vec{V}}^{\mathrm{MP}},$ $\tau_{p}^{MP}$, and $\tau_{n}^{MP}$ for the group of MP cells, and $\tau_{p}^{HP}$, $\tau_{n}^{HP}$ for the group of HP cells; black/red bars are for EGFRwt / EGFRvIII cells, respectively. The plot reveals the two distinct timescales also observed in panel C (mean ± SEM, ns = not significant, ^***^ *p* <0.001, ^****^ *p* < 0.0001. Non‐parametric ART mixed ANOVA with post‐hoc comparisons). (K) Velocity distribution of MP and HP cells in 100% EGFRwt (wt) and 100% EGFRvIII (vIII) cultures. Bold black lines indicate the mean velocity (ns= not significant, ^****^ *p* < 0.0001. Kruskal–Wallis test followed by Dunn's multiple comparisons test). (L,M) MSD plots for the separate HP and MP groups and for the entire population, shown for the EGFRvIII culture in (L) and for EGFRwt culture in (M). Data points represent experimental MSD values (mean ± SEM at each time point); solid lines represent approximate functions calculated from Equation (9), with the calculated R^2^ values >0.93.

As a quantitative measure of the cells' capacity to spread, we calculated for both cell subtypes, the mean square displacement (MSD) defined by, 〈*R* ^2^(*t*)〉, where *R*(*t*) denotes the distance traveled by a cell in a time interval *t*, and the 〈⋅⋅⋅〉 brackets indicate an averaging over all cells and all time intervals *t* in each cell's trajectory (see Methods). We found the MSD of the EGFRvIII cells to be significantly higher than that of EGFRwt cells (Figure 1A), indicating that the former cells spread over a larger area in a given time interval, consistent with our prior findings [10]. This result was in line with the higher migration velocity of the EGFRvIII cells, *V*_vIII_ = 0.87 ± 0.01 µm/min compared with *V*_WT_ = 0.78 ± 0.01 µm/min (Figure 1B). In addition, shown with the MSD plots is the so‐called cell diffusivity, *D*, defined by the long‐term asymptotic slope of the MSD curve, which characterizes the long‐term diffusive motion of the cells. This quantity is useful since it accounts for both the cellular velocity and persistence (see Methods). We found *D*_WT_ = 5.49 µ*m* ^2^/min and *D*_vIII_ = 11.38 µ*m* ^2^/min, indicating the higher dispersal capacity of these cells (Figure 1A).

To next elucidate the characteristic persistence times of cell migration, we calculated the velocity autocorrelation function (VACF) defined through: $C_{\vec{V}}\left(t\right)=\left\langle \vec{V}\left(t+t_{0}\right)\circ \vec{V}\left(t_{0}\right)\right\rangle \;$where $\vec{V}\left(t\right)$ denotes the instantaneous cell velocity, the ° symbol denotes a scalar product, and the brackets, as before, indicate an averaging over all cells and all interval starting points, *t*_0_, along a cell's trajectory. This function measures how cell velocity correlations decay over time. The analysis of this function revealed two characteristic timescales to be associated with the decay of VACF: τ_1_ ≈ 6 ± 0.4 min and τ_2_ ≈ 71 ± 6.7 min for the EGFRwt subtype (Figure 1C), and τ_1_ ≈ 7 ± 0.2 min and τ_2_ ≈ 110 ± 14.6 min for the EGFRvIII subtype (Figure 1D). The longer timescale of ≈ 70  −  100 min can also be seen with the (mild) upward bending of the MSD plots of Figure 1A on this timescale, which is a characteristic of cell persistence.

To obtain a better understanding of the origin of these distinct timescales, we more closely analyzed the cell trajectory data as follows.

#### 2.1.1 Motility of GBM Cell Subpopulations is Anisotropic in Both Velocity and Persistence

Looking at the cell trajectories, we noticed that both cell types comprised a subpopulation of roughly 15%–25% of the cells whose trajectories were significantly more anisotropic and directional than others, as shown in Figure 1E for an EGFRwt cell. To quantitatively analyze the cells' motility patterns as well as the level and origin of their anisotropy, we exploited a singular value decomposition algorithm to calculate a principal direction, $\vec{p}$, to each cell's trajectory (the sign of which was chosen to align with the mean migration direction) and quantified the corresponding cells' velocity components *V_p_* and *V_n_* with $\vec{n}$ being normal to $\vec{p}$ (see Figure 1E). Plotting the *V_p_*, *V_n_* values from all cells and all times (Figure 1F), we noticed that they scatter in an ellipse with the absolute mean values |〈*V_p_* 〉| being higher than |〈*V_n_* 〉| revealing that the cells moved faster along an inherent principal direction. By contrast, velocity components (*V_x_*, *V_y_*) along the lab frame Cartesian coordinate system showed no preferred orientation, reassuring that the noticed anisotropy was not due to external cues within the medium or the substrate. Similar anisotropic motility patterns have recently been reported with brain glioblastoma cells (U87‐MG) [13] and other cell types [17].

To examine whether the cells' persistence time along these directions was also different, we calculated for each cell the velocity autocorrelation function, $C_{\vec{V}}^{i}\left(t\right)={\left\langle \vec{V^{i}}\left(t+t_{0}\right)\circ \vec{V^{i}}\left(t_{0}\right)\right\rangle }_{t_{0}}$, and its two component contributions, $C_{p}^{i}\left(t\right)={\left\langle V_{p}^{i}\left(t+t_{0}\right)V_{p}^{i}\left(t_{0}\right)\right\rangle }_{t_{0}}$ and $C_{n}^{i}\left(t\right)={\left\langle V_{n}^{i}\left(t+t_{0}\right)V_{n}^{i}\left(t_{0}\right)\right\rangle }_{t_{0}}$ with, $C_{\vec{V}}^{i}\left(t\right)=C_{p}^{i}\left(t\right)+C_{n}^{i}\left(t\right)$. In the equations above, ‘ i ’ denotes the cell index, and the averaging ${\left\langle \text{…}\right\rangle }_{t_{0}}$ is performed over all starting points, *t*_0_, within a trajectory, as per the overlapping interval method [18]. For anisotropic motility, the velocity correlations in the $\vec{p}$ and $\vec{n}$ directions may decay on different timescales: $C_{p}^{i}\left(t\right)∼\exp(-t/\tau_{p}^{i}$), on a time scale $\tau_{p}^{i}$, and $C_{n}^{i}\left(t\right)∼\exp(-t/\tau_{n}^{i}$), on a time scale $\tau_{n}^{i}$. By fitting an exponential to the two calculated correlation functions, we extracted the corresponding timescales, $\tau_{p}^{i}\;\mathrm{and}\;\tau_{n}^{i}$ for all individual cell trajectories; representative fits are shown in the Supporting Information (Figure S2).

In Figure 1G we plotted the distributions of the collected cell persistence times, $\tau_{p}^{i}\;\mathrm{and}\;\tau_{n}^{i}$, for both the EGFRwt and the EGFRvIII subtype. Each dot corresponds to a single cell. We found the average values, ${\left\langle \tau_{p}^{i}\right\rangle }_{\mathrm{WT}}=20.6\pm 1.9\;\min$ and ${\left\langle \tau_{n}^{i}\right\rangle }_{\mathrm{WT}}=7.7\pm 0.6\;\min$ for the EGFRwt cells, and ${\left\langle \tau_{p}^{i}\right\rangle }_{\mathrm{vIII}}=27.6\pm 3.7\;\min$ and ${\left\langle \tau_{n}^{i}\right\rangle }_{\mathrm{vIII}}=10.3\pm 1.2\;\min$ for the EGFRvIII cells, revealing that the motility of both cell subtypes is not only anisotropic in their velocity along the $\vec{p}$ and $\vec{n}$ directions, but also in the persistence‐times. The values noted above indicate that on average, the cells move persistently for ∼20–30 min when migrating in the primary direction, and between 7–10 min when migrating normal to the primary direction; they also indicate that the EGFRvIII cells migrate more persistently than EGFRwt cells, and thus partially explaining their elevated spreading capacity as shown in Figure 1A.

#### 2.1.2 GBM Comprises a Highly Persistent (HP) Cell Subpopulation, Found in Both EGFRvIII‐ and EGFRwt Cells, which Largely Accounts for the Greater Spreading Capacity of the EGFRvIII Subtype

The distributions in Figure 1G revealed that both the EGFRwt and EGFRvIII populations comprised a subpopulation (15% in EGFRwt and 21% in EGFRvIII) whose motility is exceptionally directional and anisotropic since their associated persistence‐time, $\tau_{p}^{i},$ along the primary trajectory direction was 3–10‐fold higher than $\tau_{n}^{i}$. Since $\tau_{n}^{i}$ is concentrated with values ≤10min we used the value of $\tau_{p}^{i}$ to distinguish between what we called moderately persistent (MP) cells and highly persistent (HP). Cells having $\tau_{p}^{i}$ larger than a cutoff value $\tau_{p}^{\mathrm{cutoff}}=30\;\min$ were considered highly persistent, and cells with $\tau_{p}^{i}<$ $\tau_{p}^{\mathrm{cutoff}}$ were considered moderately persistent; sample cell trajectories from the two groups (MP; HP) are shown in Figure 1H,I. With the above choice of $\tau_{p}^{\mathrm{cutoff}}$, the correlation functions, $C_{p}^{\mathrm{MP}}\left(t\right)$ and $C_{n}^{\mathrm{MP}}\left(t\right)$ defined for the group of MP cells, and $C_{p}^{\mathrm{HP}}\left(t\right)$ and $C_{n}^{\mathrm{HP}}\left(t\right)$ for the group of HP cells (see Methods Equation (3)), could be well fit with a single decaying exponent each, from which we revealed the characterizing persistence times, $\tau_{p}^{MP},\tau_{n}^{MP}$ and $\tau_{p}^{HP},\tau_{n}^{HP}$, respectively (Figures S3 and S4). The cutoff value, $\tau_{p}^{\mathrm{cutoff}}=30$ min was optimized so as to obtain the best fits for the MSD profiles, whose calculation was based on those extracted persistence times (see discussion in Supporting Information and Figure S5).

The extracted values are detailed in Figure 1J for both the EGFRwt and EGFRvIII populations. For the MP cells we found a similar persistence time for migration along the trajectory's principal and normal directions ‐ for both EGFRwt and EGFRvIII subpopulations ‐ and the total VACF, $C_{\vec{V}}^{\mathrm{MP}}\left(t\right),$ could be well described by a single characteristic time, $\tau_{\vec{V}}^{\mathrm{MP}}\approx \tau_{p}^{\mathrm{MP}}\approx \tau_{n}^{\mathrm{MP}}\approx$ 6–8 min (see Figure 1J; Figures S3 and S4). In contrast, motility of the HP group was profoundly anisotropic and significantly differed between the EGFRwt and EGFRvIII subpopulations. We found, $\tau_{p}^{HP}=71\pm 8\;\min$ and $\tau_{n}^{HP}=5.1\pm 1$ min for the EGFRwt cells, and $\tau_{p}^{HP}=110\pm 14\;\min$ and $\tau_{n}^{HP}=9.4\;\pm \;1\min\;$ for EGFRvIII. The data shown in Figure 1J clearly reveal the two characteristic timescales found in the VACF plots of Figure 1C,D. One timescale mainly reflects the isotropically‐persistent motility of the MP cells, $\tau_{1}\approx \tau_{\vec{V}}^{\mathrm{MP}}\approx \tau_{p}^{\mathrm{MP}}\approx \tau_{n}^{\mathrm{MP}}\approx \tau_{n}^{\mathrm{HP}}\approx 6-8\;\min$, and the second timescale, $\tau_{2}=\tau_{p}^{\mathrm{HP}}\approx 70-110\;\min$, reflects the anisotropic persistent motion of the HP cells along a principal trajectory direction.

As will be shown below, it is the higher migration persistence of the HP cells in the EGFRvIII subpopulation that mainly accounts for the elevated MSD profile and spreading capacity of this subpopulation relative to the EGFRwt subpopulation. The distribution of cell speeds only marginally differed between the MP and HP cells and between the EGFRwt and the EGFRvIII subpopulations, with the latter's HP cells being slightly faster (Figure 1K).

Interestingly, a closer examination of the velocity distributions revealed that the velocity components distributed according to a Laplace distribution, $P\left(V_{i}\right)=\exp\left(-\frac{|V_{i}|}{b}\right)/\left(2b\right)$, and the asymptotic velocity distribution was exponential, as recently found for other cell types as well [17, 19, 20] (see Supporting Information, Laplace distributions of cell velocities section and Figure S6 for details). While the origin of this distribution is not fully understood, Wu et al. [17] proposed that it might relate to the heterogeneity of cell persistence in the population.

The MSD is mathematically related to the (stationary) velocity autocorrelation function through a general relationship, $\left\langle R^{2}\left(t\right)\right\rangle =2\int_{0}^{t}\left(t-t^{\prime }\right)C_{\vec{V}}\left(t^{\prime }\right)dt^{\prime }$. We thus further used the extracted persistence‐times to approximate the three MSD profiles associated with the MP and HP groups of cells and the entire cell population (see Methods for explicit expressions for the approximating functions). The corresponding MSD profiles and their three approximating functions are shown in Figure 1L,M. The excellent agreement of these functions to the MSD data further supports our identification of the most relevant timescales. Notably, it is the highly persistent migration along an inherently chosen primary direction of the relatively small fraction of HP cells, that makes the largest contribution to the MSD profile. This persistence is found to be larger in the EGFRvIII cell subtype and is what makes their motility more aggressive than the EGFRwt cell population.

To support the above rather complex characterization of the cell motility data, we carried out similar analysis with *in‐silico* generated cell trajectories. Simulating mixtures of anisotropic MP and HP cell trajectories with similar characteristics as experimentally found here, we were able to accurately recover the set of input values used to generate the trajectories, including the mixture composition (fraction of MP and HP cells), the moments of the velocity distribution along the two principal directions, and the distinct persistence times. The analysis and its discussion are presented in the Supporting Information (*in‐silico* section and Figure S7).

### 2.2 Co‐Culturing EGFRwt with EGFRvIII Leads to Increased Persistent Movement of the HP EGFRwt Subpopulation

We previously found that EGFRvIII cells enhance the spreading rate of EGFRwt cells [10]. To explore this phenomenon in more detail, we here quantified the effects of EGFRvIII cells on the motility parameters of the EGFRwt cells. To this end, we grew three distinct cultures of fluorescently labeled EGFRwt (Methods) and EGFRvIII cells: two co‐cultures (CC) containing 10% EGFRvIII/ 90% EGFRwt cells, or 50% EGFRvIII/ 50% EGFRwt cells (named 10%–90% and 50%–50%, respectively), as well as a homogeneous EGFRwt culture (named 100%wt), which served as a control in our study. Following 24 h of culture, we analyzed the motility characteristics of the EGFRwt cells.

**FIGURE 2:**
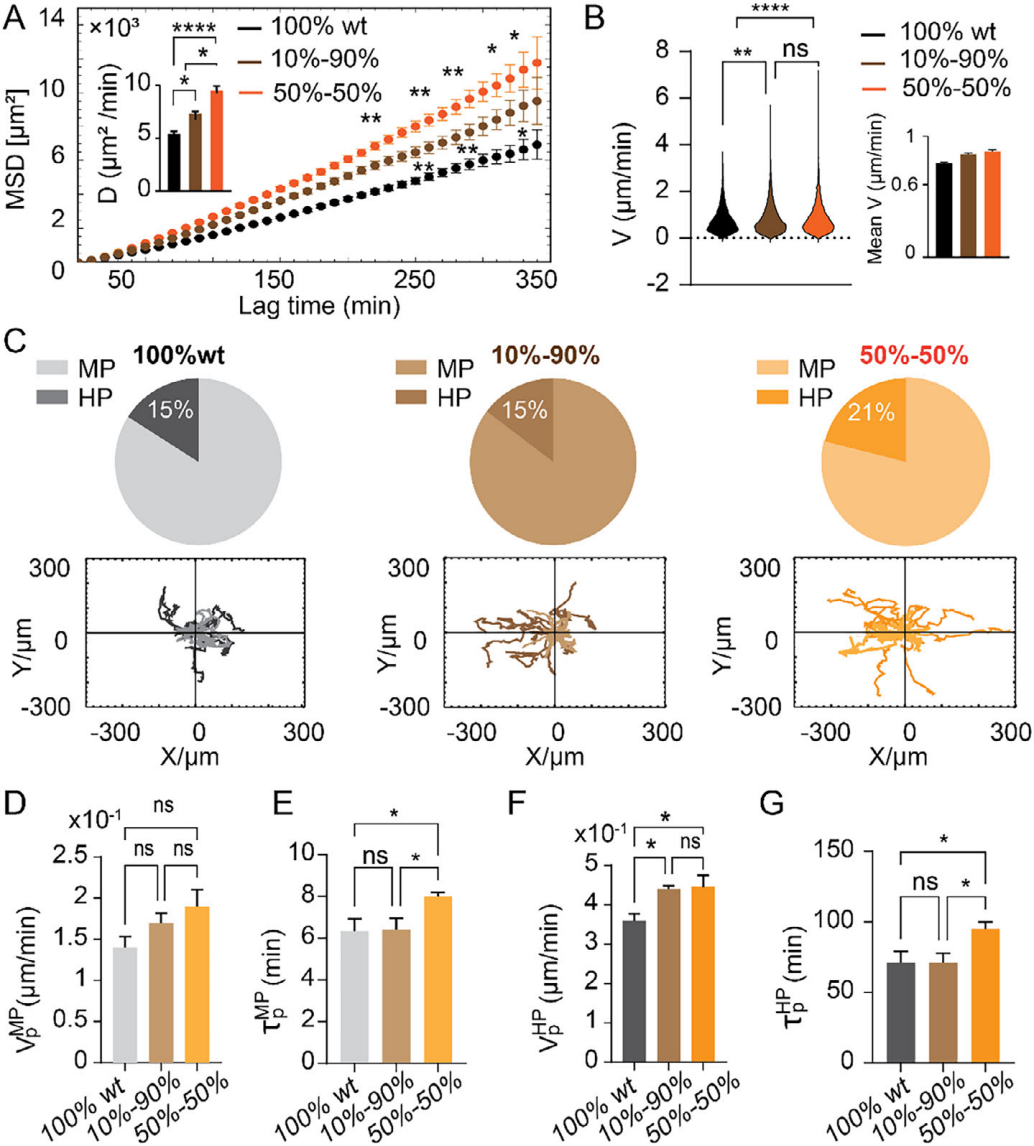
EGFRvIII cells, mainly through the highly persistent cell subpopulation, augment the spreading capacities of EGFRwt cells. (A) MSD plots calculated for EGFRwt cells, in homogeneous culture (Black, n= 138), in heterogeneous cultures (10%vIII, Brown (n=111) and 50%vIII, Orange (n=95)). (mean ± SEM at each time point. ^*^ *p* < 0.05, ^**^ *p* < 0.01. Kruskal–Wallis test with Dunn's multiple comparisons test. Only 10%–90% vs 100% and 50%–50%). Inset: Diffusivity coefficient calculated for EGFRwt cells in homogenous culture and heterogeneous cultures (mean ± s.d. ^*^ *p* < 0.05, ^****^ *p* < 0.0001. Kruskal–Wallis test with Dunn's multiple comparisons test). (B) Velocity distribution of EGFRwt cells in each group. Inset: Mean velocity calculated across all EGFRwt cells and time points, for each group. (C) EGFRwt cells’ trajectories for MP (lighter) and HP (darker) cells in each culture. Pie charts indicate the proportion of EGFRwt‐ MP and HP populations in each group. (D) Mean cellular velocity along the cell‐trajectories' principal direction for the MP group of cells in the EGFRwt subpopulation (mean ± SEM. ns= not significant. Kruskal–Wallis test with Dunn's multiple comparisons test). (E) Extracted persistence‐times for the EGFRwt MP cells in each co‐culture (mean ± SEM. ns= not significant, ^*^ *p* < 0.05. Kruskal–Wallis test with Dunn's multiple comparisons test). (F) Mean cellular velocity along the cell‐trajectories' principal direction for the HP group of cells in the EGFRwt subpopulation (mean ± SEM. ns = not significant, ^*^ *p* < 0.05. Kruskal–Wallis test with Dunn's multiple comparisons test). (I) Extracted persistence‐times for the EGFRwt HP cells in each co‐culture (mean ± SEM. ns = not significant, ^*^ *p* < 0.05. Kruskal–Wallis test with Dunn's multiple comparisons test).

Figure 2A illustrates that the mean squared displacement (MSD) and cellular diffusivity (inset panel) of EGFRwt cells consistently increased with the proportion of EGFRvIII cells in culture, suggesting that the presence of EGFRvIII cells augmented the spreading capacity of EGFRwt cells. Calculation of the velocity distribution revealed a mild but consistent increase in the mean cellular velocity (Figure 2B, right panel). This pattern is also evident in the representative trajectories shown in Figure 2C.

To obtain a better understanding of the impact of EGFRvIII cells on EGFRwt motility, we conducted a characterization of cell trajectories similar to that performed with the homogenous EGFRwt and EGFRvIII cultures reported in the preceding section.

Consequently, we computed the VACFs for each individual cell in the control and the two co‐cultures. As with the homogeneous cultures discussed previously, our analysis here revealed the existence of a highly persistent (HP) EGFRwt cell subpopulation in the control (100%wt) and the two co‐cultures (Figure S8A–C). Using the same cutoff value, $\tau_{p}^{\mathrm{cutoff}}=30\;\min$, we sorted the EGFRwt cells into MP and HP subpopulations and calculated their ensemble‐averaged motility characteristics (Figure S8D,E), particularly their mean velocities, $V_{p}^{MP}$ and $V_{p}^{HP}$ (Figure 2D,F), persistence‐times, $\tau_{p}^{MP}\mathrm{and}$ $\tau_{p}^{HP}$ (Figure 2E,G), fractions of cells in each subpopulation, *N_MP_*/*N*, and *N_HP_*/*N* (Figure 2C, upper panels), and the MSD profiles, 〈*R* ^2^(*t*)〉_*MP*_ and 〈*R* ^2^(*t*)〉_*HP*_ (Figure S8A,B). The presence of 10% EGFRvIII cells resulted in a modest increase (∼8%) of EGFRwt HP cells’ mean velocity (Figure 2F) with no effect on persistence (Figure 2G), suggesting that in this percentage of EGFRvIII cells, the effects on the MSD arose mainly from the effect on cellular velocity.

In contrast, when the EGFRvIII percentage was raised to 50%, we found in addition to a ∼17% increase of EGFRwt HP cell velocity (Figure 2F), a notable increase in persistence times of both the MP‐ (∼23%) and HP‐ (∼58%) subpopulations (Figure 2E,G), as well as in the fraction of HP cells in culture (Figure 2C).

These data indicate that the presence of EGFRvIII cells in co‐culture both enriches the EGFRwt population with a larger fraction of HP cells, as well as induces a more aggressive migratory phenotype of the two (MP and HP) groups of cells. Although our data do not provide direct evidence of MP‐to‐HP state transitions, the observed changes suggest that EGFRvIII signaling directs MP cells toward a more HP‐like motility phenotype in addition to enhancing the motility of the existing HP group, thereby augmenting the overall dissemination of the EGFRwt population as reported previously in [10].

### 2.3 Src Inhibition Decreases the Spreading Capacity of EGFRwt Cells by Attenuating Their Persistence

**FIGURE 3:**
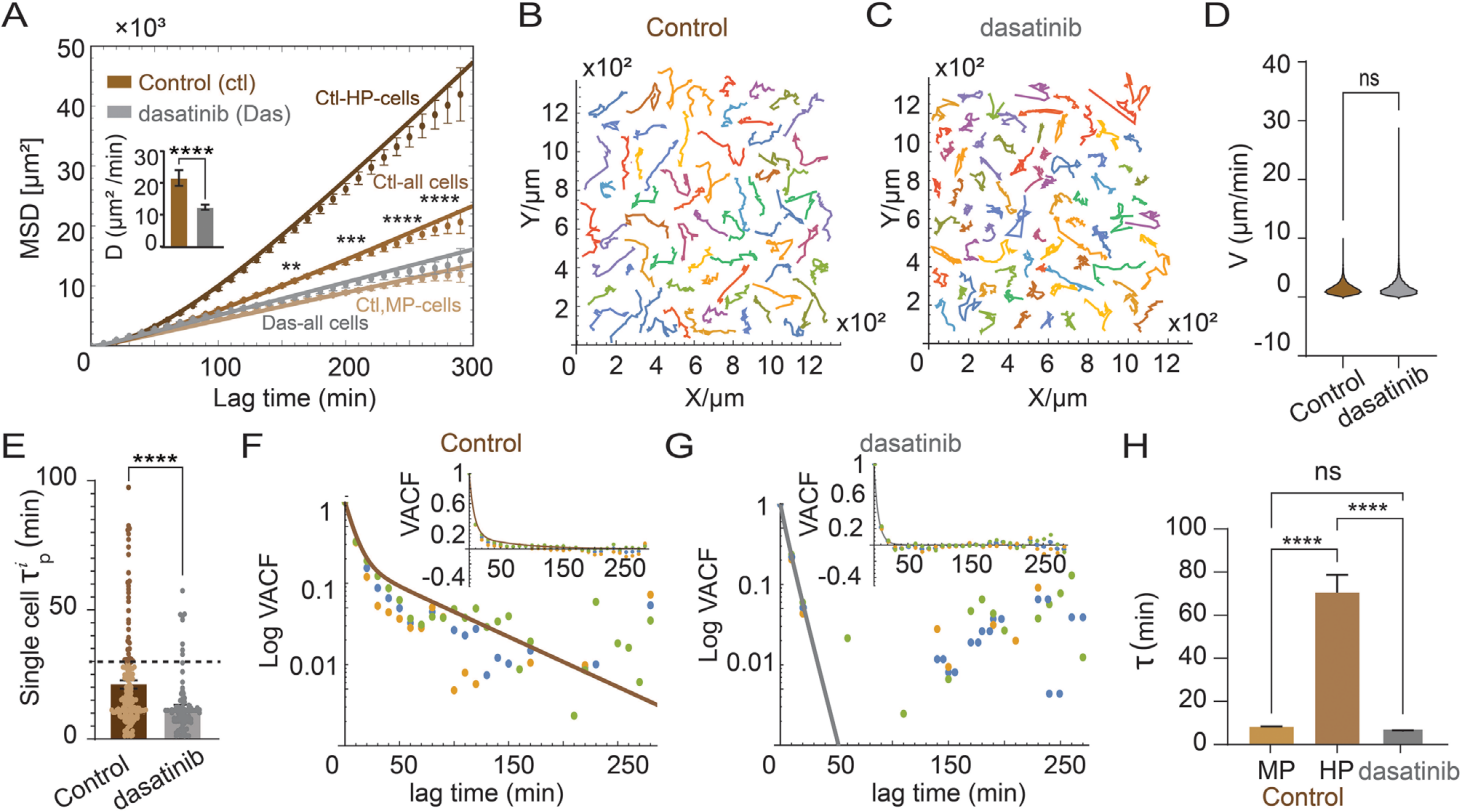
Src inhibition suppresses EGFRwt cells' increased spreading in co‐culture, mostly through the elimination of HP cells. (A) MSD plots calculated for EGFRwt cells, in heterogeneous culture (10%vIII, control, brown, n =150) and when treated with dasatinib (Src inhibitor, 200 nm, gray, n= 83). Solid lines are approximate functions calculated from Equation (9) with R^2^ values >0.95. The statistical significance symbols denote the comparison between control (Ctl) and dasatinib (Das) MSD curves for all cells (mean ± SEM.^**^ *p* < 0.01, ^***^ *p* < 0.001, ^****^ *p* < 0.0001. Mann–Whitney test (per‐time‐point) with multiple‐comparison correction). (B,C) EGFRwt cells’ trajectories (B) in control and (C) in dasatinib. (D) Cell velocity distribution of U87EGFRwt cells in each group (ns=not significant. Mann–Whitney U test (two‐tailed)). (E) Distributions of single cell persistence times along the cell trajectories' principal direction, for both EGFRwt cells in control (brown) and dasatinib (gray). Each dot represents an individual cell (^****^ *p* < 0.0001. Mann–Whitney U test). The dashed line marks the 30‐min cutoff value used to sort the cells into MP and HP cells. (F,G) Velocity autocorrelation function (VACF) for (F) control and (G) dasatinib EGFRwt cells as a function of lag time shown in long‐linear scale; solid lines are calculated from Equation (4) with calculated R^2^ values >0.95. The inset panels in (F,G) display the VACF in linear scale. (H) Extracted persistence times (mean ± SEM. ns=not significant, ^****^ *p* < 0.0001. Kruskal–Wallis test followed by Dunn's multiple comparisons test) $\tau_{\vec{V}}^{\mathrm{MP}}$ for the MP cells, and $\tau_{p}^{HP}$ for the HP cells in the control group, and $\tau_{\vec{V}}$ for all cells in the dasatinib‐treated group.

EGFRwt and EGFRvIII cells have been found to interact through the Src pathway, consequently modulating the augmented infiltrative characteristics of EGFRwt cells. In our prior study, we demonstrated that the inhibition of Src significantly diminished the increased spreading of EGFRwt cells when co‐cultured with the EGFRvIII population [10]. In that work, we also established that the concentration of dasatinib used here (200 nM) was non‐cytotoxic in a separate viability assay over the duration of the experiment, confirming that the change in motility was a result of functional reprogramming and not selective cell death. Furthermore, we confirmed the selective role of Src using siRNA‐mediated genetic knockdown of Src in the EGFRwt cells, which phenocopied the effect of dasatinib [10]. However, a quantitative examination of the effects on specific characteristics of cell motility has not been performed. Consequently, we co‐cultured EGFRwt cells with 10% EGFRvIII cells, a ratio selected to represent the clinically documented proportion of the EGFRvIII subpopulation within heterogeneous GBM tumors [21, 22], in the presence of the anti‐ Src drug (dasatinib) and analyzed the Src‐inhibition effect on EGFRwt cell motility in CC. Dasatinib treatment led to a significant decrease in the MSD profile and cell diffusivity (Figure 3A), resulting in more erratic and compacted cell trajectories (Figure 3B,C).

Despite the lack of impact on cell velocity due to Src inhibition (Figure 3D) and the maintenance of the velocity anisotropy (Figure S9), the examination of both individual cell persistence times (Figure 3E) and the ensemble‐averaged velocity autocorrelation function (VACF) (Figure 3F,G) demonstrated a significant influence on cell persistence. Following Src inhibition, there were few HP cells (Figure 3E), and the ensemble‐averaged VACF exhibited a single timescale, $\tau_{\vec{V}}=7.65\pm 0.57$ min (Figure 3G,H), similar to the extracted timeframe of MP cells in the control (untreated) group (Figure 3H). These results underscore the significance of Src activation in augmenting cell persistence during migration and facilitating their dispersion across long distances.

### 2.4 The Role of the Local Microenvironment in EGFRwt/EGFRvIII Cell Communication

A prior work demonstrated that soluble molecules released by isolated homogeneous EGFRwt glioblastoma cell pairs can establish a local microenvironment that affects the direction of cell migration [23]. Cells originally located less than about 200 µm apart had a tendency to migrate away from one another, whereas cell pairs commencing more than approximately 200 µm apart traveled toward one another, ultimately stabilizing at a preferred distance. The directional responses were demonstrated to be governed by both the initial cell‐cell distance and the concentrations of released soluble substances [23].

**FIGURE 4:**
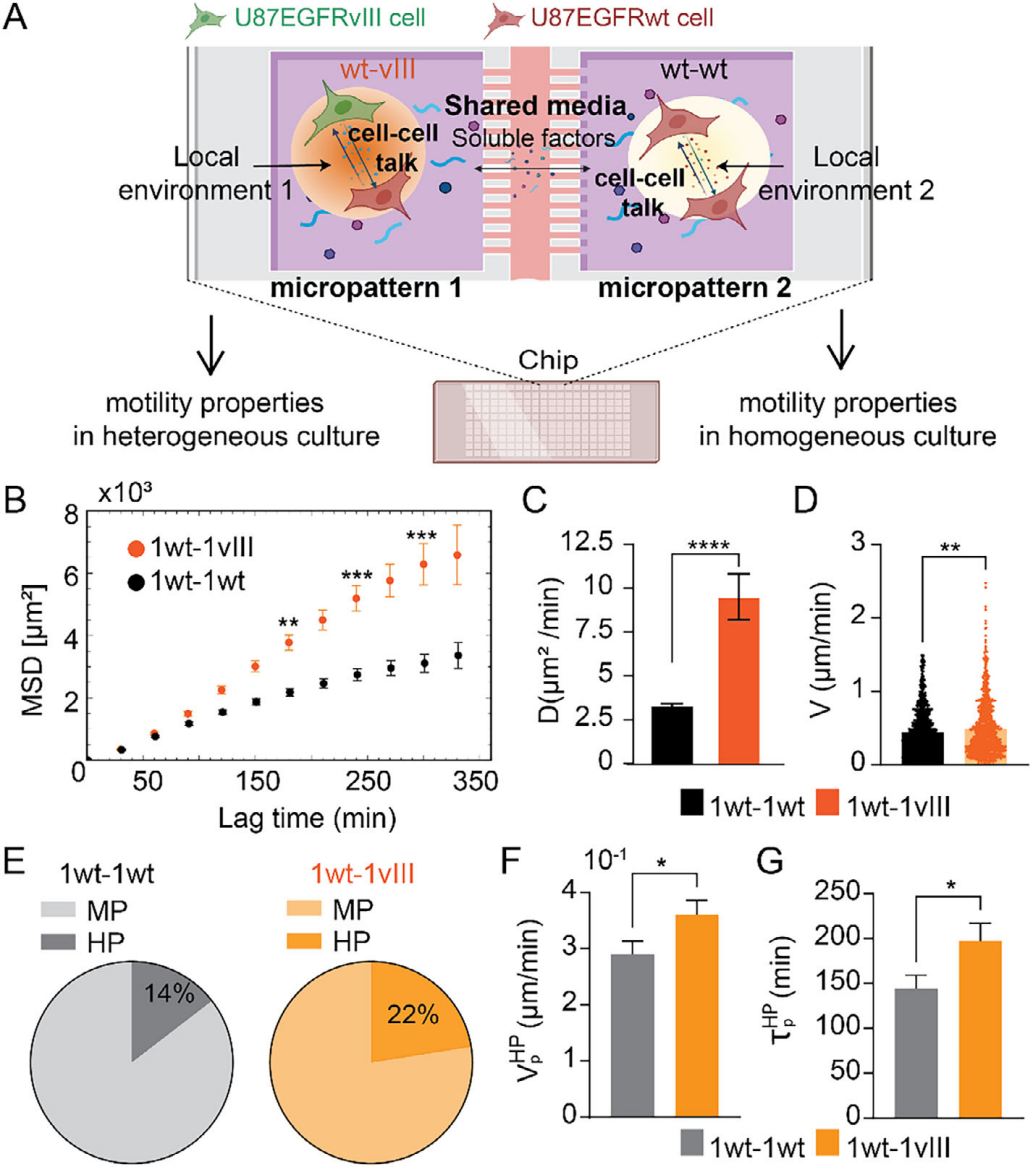
The local microenvironment from EGFRwt/EGFRvIII cell communication plays a major part in U87EGFRwt cell motility, increasing cell velocity, persistence, and diffusivity. (A) schematic overview of the experiment aim. (B) Mean Squared Displacement of EGFRwt calculated for EGFRwt‐EGFRwt pairs (black, n = 103) and EGFRvIII‐EGFRwt pairs (orange, n = 80), (mean ± SEM, ^**^ *p* < 0.01, ^***^ *p* < 0.001, Mann–Whitney U test (two‐tailed)). (C) Diffusivity coefficient of EGFRwt calculated for wt‐wt and wt‐vIII cell pairs. (mean ± s.d., ^****^ *p* < 0.0001. Mann–Whitney U test (two‐tailed)). (D) Cell velocity calculated for EGFRwt in wt‐wt and wt‐vIII cell pairs. Columns represent averages of velocities of all cells (EGFRwt) at final time point in each group (mean ± SEM., ^****^ *p* < 0.0001. Mann–Whitney U test (two‐tailed)). (E) Pie charts indicating the proportion of EGFRwt‐ MP and HP populations in each culture. (F) Mean velocity along the principal trajectory direction for HP EGFRwt‐ cells in each group (mean ± SEM, ^*^ *p* < 0.05, Mann–Whitney U test (two‐tailed)). (G) Extracted persistence times for HP cells in each group (mean ± SEM, ^*^ *p* < 0.05, Mann–Whitney U test (two‐tailed)).

To investigate how cell‐cell contact between homogeneous (EGFRwt/EGFRwt) and heterogeneous (EGFRwt/EGFRvIII) cell pairings influences migration, we used a micropatterned chip (see SI, chip analysis). This chip contained adhesive circular micropatterns designed to trap a limited number of cells and physically isolate them from neighboring groups. We utilized micropatterns with a 225 µm diameter, spaced 325 µm center‐to‐center, creating a minimum 100 µm gap between the edges of adjacent chambers. This design allowed us to physically restrict cells to specific micropatterns while still enabling intercellular communication through the diffusion of soluble chemicals across the shared culture medium (Figure 4A). We calibrated the system such that most of the micropatterns had two cells (see Methods and Figure 4A), which can be homogeneous EGFRwt cells or heterogeneous EGFRwt‐EGFRvIII pairings. Time‐lapse images were acquired every 30 min (rather than the 10‐min interval used previously) to minimize phototoxicity and light sensitivity in the confined chip environment. Since for the MP cell subpopulation we found a persistence time on the order of (∼6–8 min in bulk culture), the 30‐min temporal resolution was insufficient to capture their short‐lived, irregular migratory patterns. Thus, in the micropattern system, we focused on the more persistent HP cells.

Despite the shared culture medium allowing diffusion of soluble factors between micropatterns, our analysis revealed a significant impact of local cell‐cell interactions on migratory behavior. We specifically found that EGFRwt cells exhibited enhanced spreading capacity only when heterogeneously coupled with EGFRvIII cells within the same micropattern, in contrast to homogeneous pairings of EGFRwt cells, which did not show this increased spreading. Indeed, measurements of EGFRwt cells' motility characteristics in heterogeneous pairings showed enhanced Mean Squared Displacement (MSD) (Figure 4B), increased cell diffusivity (Figure 4C), and elevated overall migration velocity (Figure 4D) compared to the homogenous ones. Furthermore, the proportion of highly persistent (HP) cells increased in the heterogeneous culture (Figure 4E), characterized by an enhanced cell velocity (Figure 4F) and persistence (Figure 4G) when directly compared to homogeneous pairings with other EGFRwt cells.

These findings provide critical evidence that the EGFRvIII‐mediated enhancement of EGFRwt cell motility is not merely a bulk‐level effect but rather a result of highly localized, short‐range cell‐cell communication that establishes a functional “microenvironment” directly between interacting cells, even in the presence of broader soluble signals on a global scale.

## 3 Discussion

Glioblastoma (GBM) remains one of the most lethal and infiltrative primary brain tumors [24], with near‐universal recurrence due to the dispersion of cancer cells into the surrounding brain tissue, rendering complete surgical resection impossible. A key driver of this aggressive phenotype is the complex interplay between different subpopulations of cancer cells within the tumor.

This study provides a detailed biophysical characterization of how a small subpopulation of GBM cells expressing the mutant EGFRvIII [15] reprograms the larger subpopulation of EGFRwt‐expressing cells to adopt a more aggressive and persistent migratory behavior, thereby driving tumor dissemination.

Our study began by quantitatively defining the baseline migratory behaviors of EGFRvIII and EGFRwt GBM cells. We confirmed that EGFRvIII cells are intrinsically more aggressive, demonstrating significantly higher mean velocity and greater dispersal over time (Mean Squared Displacement ‐ MSD) compared to EGFRwt cells. Our analysis uncovered a deeper layer of complexity: the motility of both cell types is anisotropic, with cells preferentially moving along a self‐determined primary axis with greater speed and persistence. This intrinsic directionality is a key feature of their invasive potential.

One of the significant findings of the study is the identification of two distinct kinetic subpopulations within both EGFRwt and EGFRvIII cultures: a majority of “moderately persistent” (MP) cells and a smaller, but significantly influential, subpopulation of “highly persistent” (HP) cells. These HP cells, characterized by their remarkably persistent and sustained trajectories, are the primary contributors to the enhanced spreading capacity observed in the EGFRvIII population. The existence of these HP cells provides a mechanistic explanation for the disproportionate impact of a minority cell population on the overall tumor's aggressive behavior. While both cell types contain HP cells, the HP subpopulation within the EGFRvIII culture is more persistent than its EGFRwt counterpart, accounting for the increased aggressiveness of EGFRvIII‐dominant tumors.

Building on this, our co‐culture experiments revealed the central mechanism of tumor progression: intercellular communication. When cultured with EGFRvIII cells, EGFRwt cells exhibited a dramatic shift in behavior, adopting a more aggressive migratory profile. The presence of EGFRvIII cells specifically amplified the aggressive characteristics of the EGFRwt HP subpopulation. This suggests that EGFRvIII cells enhance the migratory capabilities of the most invasive EGFRwt cells, effectively transforming the entire tumor into a more potent infiltrative machine. This finding clearly illustrates how intratumoral heterogeneity actively drives invasion, where aggressive subclones can modulate the phenotype of their less aggressive neighbors.

Furthermore, our work identifies the Src signaling pathway as the molecular conduit for this cellular crosstalk. In line with previous findings [10], we demonstrated that inhibiting Src with the FDA‐approved drug dasatinib effectively reverses the pro‐migratory reprogramming of EGFRwt cells. This conclusion is strongly supported by complementary experiments using siRNA‐mediated genetic knockdown of Src, which yielded identical anti‐migratory effects [10]. Critically, the inhibitory effect on motility was observed at non‐cytotoxic concentrations (200 nM) of dasatinib, confirming that the change in phenotype is due to functional repression of the Src pathway in the EGFRwt cells rather than selective cell death [10]. Dasatinib treatment profoundly impacted migration persistence, causing a near‐complete elimination of the HP phenotype among EGFRwt cells in co‐culture. This effect converted the highly directed movement of HP cells into a more random, less effective pattern of dispersal, drastically reducing their overall MSD. These findings position Src as a critical regulator of migratory *persistence*, a key biophysical parameter that, as our data shows, is paramount for aggressive dissemination.

Finally, through the use of specialized micropatterned chips, we demonstrated that this Src‐mediated communication is highly dependent on the local microenvironment. Enhanced motility of EGFRwt cells was observed only when they were in close contact with an EGFRvIII cell within the same isolated micro‐chamber. This suggests that the signaling mechanism, likely involving a combination of secreted factors and physical cues, operates over a short range. The mechanism likely hinges on the crosstalk factors, IL‐6 and HGF, previously shown to activate Src and FAK in EGFRwt cells [10]. The localized requirement suggests that the secreted factors must act quickly and locally to modulate focal adhesion dynamics, a key process regulated by Src, to facilitate the efficient detachment and motility required for the persistent migration pattern. This localized interaction is sufficient to increase the velocity, persistence, and diffusivity of the neighboring EGFRwt cell, highlighting the critical role of immediate cell‐to‐cell proximity in dictating invasive behavior.

In conclusion, this study deciphers a critical mechanism of GBM infiltration by bridging molecular signaling with a detailed quantitative characterization of cell motility. We demonstrate that aggressive EGFRvIII cells induce less aggressive EGFRwt cells to migrate faster and, more importantly, with greater persistence. This process is driven by a small, highly persistent subpopulation and is mediated by paracrine, Src‐dependent signaling that requires a localized microenvironment. Crucially, the translational relevance of this mechanism was previously confirmed using patient‐derived GBM cell lines (3731 line) harboring the EGFRvIII mutation, which showed a significant reduction in infiltrative spreading upon dasatinib treatment in a 3D model [10]. Furthermore, the enhanced infiltrative phenotype of the mixed EGFRvIII/EGFRwt population was also validated in an in vivo mouse model [10].

By identifying migratory persistence, regulated by the Src pathway, as a key therapeutic vulnerability, our findings offer a compelling rationale for targeting this pathway to disrupt the cellular communication that fuels the invasion of glioblastoma into the brain.

## 4 Materials and Methods

### 4.1 Cell Culture

U87EGFRwt and U87EGFRvIII cell lines were engineered from the U87 human glioblastoma cell line [16] to overexpress EGFRwt (U87EGFRwt) or EGFRvIII (U87EGFRvIII ‐LacZ), with levels of 0.5 × 10^6^–1 × 10^6^ receptors per cell that were found to be consistent with amplified EGFR levels frequently found in GBM tumors prepared from patient material [7]. The Technion Institute's Genomic Center validated both cell lines (Haifa). The medium composed of DMEM (high glucose), 10% FCS, 1% L‐glutamine, 100U/ml Penicillin and 100mg/ml Streptomycin antibiotic.

mCherry U87EGFRwt cells were generated as described in [10]

In CC experiments, either 90% of U87EGFRwt and 10% of U87EGFRvIII cells or 50% of U87EGFRwt and 50% of U87EGFRvIII cells were seeded and incubated in a single plate.

### 4.2 Antibodies and Reagents

Dasatinib (CAY11498‐50 mg), Laminin (LaminStemTM 521, BI #05‐753‐1F, Biological Industries), NLS GFP‐Luciferase virions (CellLightTM BacMam 2.0, Thermo Fisher, C10602), NLS RFP‐Luciferase virions (CellLightTM BacMam 2.0, Thermo Fisher, C10603).

### 4.3 Transfection with GFP and RFP

In a 24‐well plate, U87EGFRwt cells and U87EGFRvIII cells were seeded at 70% confluence as homogeneous cultures. The following day, U87EGFRwt and U87EGFRvIII cells were labeled with either NLS GFP‐Luciferase virions or NLS RFP‐Luciferase virions. For more details, see the Supporting Information.

### 4.4 Bulk Assay

Two experiments were conducted with similar seeding conditions: U87EGFRwt cells and U87EGFRvIII cells were co‐seeded in 24‐well plates coated with 5 µg/mL laminin overnight at 4°C.

Experiment 1: 100%wt, 100%vIII, CC (10%–90%,) CC (50%–50%) Conditions: U87EGFRwt cells were transfected with RFP stained with Hoechst 33342 at a concentration of 1:1000, whereas EGFRvIII cells were transfected with GFP.
Experiment 2: Co‐culture (10%–90%) with dasatinib Treatment: The same seeding density was used for the co‐culture (CC) condition; however, this time, mCherry‐labeled U87EGFRwt cells were used to track their movement. The next day, cells were either left untreated (control) or treated with 200 nm dasatinib. The cells were tracked for 6 h post‐treatment using time‐lapse imaging.

### 4.5 Chip Assay

2 × 10^4^ mcherry‐labeled U87EGFRwt cells and 2 × 10^4^ unlabeled U87EGFRvIII cells were seeded onto the chip and incubated for 6 h. The suspended cells were removed, and the chip containing adherent cells was placed in the microscope's incubator chamber for live cell imaging for 6 h. During the computational analysis, heterogeneous and homogeneous U87EGFRwt pairs, as well as single cells, were measured in the same micro‐well. For more details, see the Supporting Information.

### 4.6 Time‐Lapse Imaging

U87EGFRwt cells were typically labeled with GFP, whereas U87EGFRvIII cells were labeled with RFP. 3 × 10^4^ cells were seeded into a 24well plate, either as a homogeneous culture of U87EGFRwt cells only or as a CC of 10%–90% (U87EGFRvIII:U87EGFRwt) or 50%–50%. (U87EGFRvIII: U87EGFRwt). The cells were then incubated overnight before being imaged every 10 min (bulk assay) and 30 min (chip assay) for 6 h.

### 4.7 Image and Data Analysis

Cell tracking was done using the NIS‐element software, where the x, y coordinates of each cell have been collected at time intervals, Δ*t*, of 10 min, for a total duration of (*N* − 1)Δ*t* = 350 min, with *N* = 36 the number of time points in each trajectory. The trajectory data was then analyzed with Wolfram Mathematica software. The following quantities have been calculated.

#### 4.7.1 Cell Velocity

With the cell position at time point *t_i_* = *i*Δ*t*, denoted by $\vec{r}\left(t_{i}\right)$, the instantaneous cell velocity was approximated by, $\vec{V}\left(t_{i}\right)=\left(\vec{r}\left(t_{i+1}\right)-\vec{r}\left(t_{i}\right)\right)/\Delta t$. Velocity distributions, their moments, and correlation functions were then calculated.

#### 4.7.2 Principal Directions for Individual Cell Trajectories

With each cell trajectory, we associated a principal direction, $\vec{p}$, and a normal direction $\vec{n}$. Trajectories were first centered at their average position via the transformation 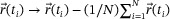. Then, a *N* × 2 trajectory matrix, M, was defined for each cell from the x, y coordinates at the *N* trajectory time points. We then used the Mathematica SingularValueDecomposition[M] function to obtain $\vec{p}$ and $\vec{n}$. Since the algorithm does not distinguish between the initial and final time points of a trajectory, the sign of $\vec{p}$ was then adjusted to match the mean direction of movement, $1/\left(N-1\right)\sum_{i=1}^{N-1}\vec{V}\left(t_{i}\right)$.

#### 4.7.3 Single‐Cell Velocity Autocorrelation Functions and Associated Persistence Times

Velocity autocorrelation functions were calculated for individual cell trajectories as follows:

(1) $$C_{\vec{V}}^{i}\left(t=k\Delta t\right)=\frac{1}{N-k-1}\sum_{j=0}^{N-k-2}\vec{V^{i}}\left(t_{j}\right)\circ \vec{V^{i}}\left(t_{j+k}\right)$$

Here *t* = *k*Δ*t* is a time lag between two points on a trajectory, and *k* is the lag index, varying from 0 to *N* − 2. Since the velocity correlations along the $\vec{p}$ and $\vec{n}$ directions decayed on different timescales we separately calculated the two components, $C_{p}^{i}\left(t\right)$ and $C_{n}^{i}$, defined through:

(2) $$C_{\alpha }^{i}\left(t=k\Delta t\right)=\frac{1}{N-k-1}\sum_{j=0}^{N-k-2}V_{\alpha }^{i}\left(t_{j}\right)V_{\alpha }^{i}\left(t_{j+k}\right);\alpha =\left(p,n\right)$$

with $C_{p}^{i}\left(t\right)+C_{n}^{i}\left(t\right)=C_{\vec{V}}^{i}\left(t\right).$ To extract the associated persistence times $\tau_{p}^{i}\pm SE$ and $\tau_{n}^{i}\pm SE$ (with SE the standard error), we used Mathematica's non‐linear least‐squares fit algorithm to fit the correlation data with single decaying exponents.

#### 4.7.4 Ensemble‐Averaged Velocity Autocorrelation Functions and Associated Persistence Times

As our data revealed the existence of a subpopulation of cells with exceptionally large $\tau_{p}^{i}$, we used a cutoff value, $\tau_{p}^{\mathrm{cutoff}}=30\;\min$, to sort the cells into moderately persistent (MP) and highly persistent (HP) cells. MP cells had $\tau_{p}^{i}<\tau_{p}^{\mathrm{cutoff}}$ and HP cells had $\tau_{p}^{i}\geq \tau_{p}^{\mathrm{cutoff}}$. Denoting by G = (HP, MP) the group of cells averaged upon, and by *N_G_* the number of cells in that group, the associated ensemble‐averaged correlation functions were calculated via:

(3) $$\begin{array}{c} C_{\vec{V}}^{G}\left(t\right)=C_{p}^{G}\left(t\right)+C_{n}^{G}\left(t\right);\;\;\;C_{p,n}^{G}\left(t\right)=\frac{1}{N_{G}}\sum_{i=1}^{N_{G}}C_{p,n}^{i}\left(t\right);\;\;\;\;\;\;\;\\ with\;G=\left(\mathrm{HP},\mathrm{MP}\right) \end{array}$$

With $\tau_{p}^{\mathrm{cutoff}}=30\;\min$, the four separate components $C_{p,n}^{G}\left(t\right)$ could be well fit with a single decaying exponent each, characterized by the associated persistence times $\tau_{p,n}^{G}\pm SE$. For the MP group we found $\tau_{p}^{MP}\approx \tau_{n}^{MP}$ and the velocity autocorrelation function $C_{\vec{V}}^{\mathrm{MP}}\left(t\right)$ could as well be fit with a single exponent, $∼\exp(-t/\tau_{\vec{V}}^{\mathrm{MP}})$, with $\tau_{\vec{V}}^{\mathrm{MP}}$ denoting the associated persistence time. With the calculated fractions of MP and HP cells, χ_*MP*_ = *N_MP_*/*N_All_* and χ_*HP*_ = *N_HP_*/*N_All_*, the computed mean square velocities, 〈*V* ^2^〉_*MP*_, ${\left\langle V_{p}^{2}\right\rangle }_{HP}$, ${\left\langle V_{n}^{2}\right\rangle }_{HP}$, and the extracted persistence times, $\tau_{\vec{V}}^{\mathrm{MP}}$, $\tau_{p}^{HP}$, $\tau_{n}^{HP}$, we used the following equation to approximate the calculated VACF data:

(4) $$\begin{array}{c} C_{\vec{V}}^{\mathrm{Approx}}\left(t\right)=\chi_{\mathrm{MP}}{\langle V^{2}\rangle }_{\mathrm{MP}}\exp\left(-t/\tau_{\underset{V}{\to }}^{\mathrm{MP}}\right)+\\ \chi_{\mathrm{HP}}\left[{\langle V_{p}^{2}\rangle }_{\mathrm{HP}}\exp\left(-t/\tau_{p}^{\mathrm{HP}}\right)+{\langle V_{n}^{2}\rangle }_{\mathrm{HP}}\exp\left(-t/\tau_{n}^{\mathrm{HP}}\right)\right] \end{array}$$

This function is drawn by solid lines in Figure 1C,D along with the calculated VACF data for the EGFRwt and EGFRvIII subpopulations. The choice, $\tau_{p}^{\mathrm{cutoff}}=30\;\min$, was found to obtain the best fits for the ensemble‐averaged correlation functions and the MSD profiles discussed below, and was also supported by computer simulations as described in the Supporting Information.

#### 4.7.5 Mean Square Displacement (MSD)

The MSD is defined through 〈*R* ^2^(*t*)〉 where R(t) is the distance traveled by a cell in a time interval *t* and the brackets indicate an average over all cells and all time intervals *t* within a trajectory. For a discrete‐time trajectory, denoting by $|\vec{r^{i}}\left(t_{j+k}\right)-\vec{r^{i}}\left(t_{j}\right)|$ the distance traveled by cell i in a time interval *t*_*j* + *k*_ − *t_j_* = *k*Δ*t*, the MSD is given by:

(5) $$\begin{array}{ccc} & & {\langle R^{2}\left(t=k\Delta t\right)\rangle }_{G}\\ & = & \frac{1}{N_{G}}\times \frac{1}{N-k-1}\sum_{i=1}^{N_{G}}\sum_{j=0}^{N-k-2}{\left[\vec{r^{i}}\left(t_{j+k}\right)-\vec{r^{i}}\left(t_{j}\right)\right]}^{2} \end{array}$$

G = (MP, HP, All) where again, G denotes the group of cells averaged upon (All implying all cells), *N_G_* the number of cells in that group, *k* the time gap index, and *N* the number of time points in a trajectory. The MSD is generally related to the velocity autocorrelation function [25]. For a process with the velocity autocorrelation function of the form:

(6) $$C_{\vec{V}}\left(t\right)=\sum_{\alpha }a_{\alpha }\exp\left(-t/\tau_{\alpha }\right)$$

one finds the following expression for the MSD:

(7) $$\langle R^{2}\left(t\right)\rangle =2\sum_{\alpha }a_{\alpha }\tau_{\alpha }\left[t-\tau_{\alpha }\left(1-\exp\left(-t/\tau_{\alpha }\right)\right)\right]$$

For a homogeneous population of cells, showing anisotropic motility with distinct velocities and persistence times along the principal and non‐principal trajectory directions, one finds the following approximating expression for the MSD:

(8) $$\begin{array}{ccc} {\langle R^{2}\left(t\right)\rangle }_{G} & = & 2\left\{{\langle V_{p}^{2}\rangle }_{G}\tau_{p}^{G}\left[t-\tau_{p}^{G}\left(1-\exp\left(-t/\tau_{p}^{G}\right)\right)\right]\right.\\ & & \left.+{\langle V_{n}^{2}\rangle }_{G}\tau_{n}^{G}\left[t-\tau_{n}^{G}\left(1-\exp\left(-t/\tau_{n}^{G}\right)\right)\right]\right\} \end{array}$$

with, G, relating as before, to either the HP or the MP cell subpopulations. However, in the case of the MP cells we found only a minor difference between the two extracted persistence times and consequently both the autocorrelation and the MSD functions could as well be fit upon using a single characteristic persistence time, $\tau_{\vec{V}}^{\mathrm{MP}}$. For a cell population comprising a fraction, χ_*MP*_ = *N_MP_*/*N_All_* of MP cells, and a fraction, χ_*HP*_ = *N_HP_*/*N_All_* of HP cells ‐ the former of which associated with a mean square velocity, 〈*V* ^2^〉_*MP*_, and a single characteristic persistence time, $\tau_{\vec{V}}^{\mathrm{MP}}$, and the latter of which associated with a mean square velocity, 〈*V* ^2^〉_*HP*_ and two characteristic persistence times, $\tau_{p}^{HP}$ and $\tau_{n}^{HP}$ ‐ one finds the following approximating expression for the MSD from Equation (6):

(9) $$\begin{array}{ccc} \langle R^{2}\left(t\right)\rangle & = & 2\left\{\chi_{\mathrm{MP}}{\langle V^{2}\rangle }_{\mathrm{MP}}\tau_{\vec{V}}^{\mathrm{MP}}\left(1-\exp\left(-t/\tau_{\vec{V}}^{\mathrm{MP}}\right)\right)\right.\\ & & \;\left.+\chi_{\mathrm{HP}}{\langle V_{p}^{2}\rangle }_{\mathrm{HP}}\tau_{p}^{HP}\left[t-\tau_{P}^{HP}\left(1-\exp\left(-1/\tau_{P}^{HP}\right)\right)\right]\right.\\ & & \;\left.+\chi_{\mathrm{HP}}{\langle V_{n}^{2}\rangle }_{\mathrm{HP}}{\tau n}^{\mathrm{HP}}\left[t-{\tau n}^{\mathrm{HP}}\left(1-\exp\left(-t/{\tau n}^{\mathrm{HP}}\right)\right)\right]\right\} \end{array}$$

With the calculated fractions of HP and MP cells, the computed mean square velocities, 〈*V* ^2^〉_*MP*_, ${\left\langle V_{p}^{2}\right\rangle }_{HP}$, ${\left\langle V_{n}^{2}\right\rangle }_{HP}$, and the extracted persistence times, $\tau_{\vec{V}}^{\mathrm{MP}}$, $\tau_{p}^{HP}$, $\tau_{n}^{HP}$, we have used Equation (9) to obtain approximate profiles for our calculated MSD data; these are shown in our MSD plots along with the measured MSD data.

#### 4.7.6 Cell Diffusivity

As the cell diffusivity, *D*, is defined as 1/(2*n*) the asymptotic long‐term slope of the MSD profile (with *n* the system's dimensionality), it can be obtained from Equations (6) and (7) in the limit t→∞ as follows:

(10) $$2D_{G}={\langle V_{p}^{2}\rangle }_{G}\tau_{p}^{G}+{\langle V_{n}^{2}\rangle }_{G}\tau_{n}^{G}$$

(11) $$2D=\chi_{\mathrm{MP}}{\langle V^{2}\rangle }_{\mathrm{MP}}\tau_{\vec{V}}^{\mathrm{MP}}+\chi_{\mathrm{HP}}\left[{\langle V_{p}^{2}\rangle }_{\mathrm{HP}}\tau_{p}^{\mathrm{HP}}+{\langle V_{n}^{2}\rangle }_{\mathrm{HP}}\tau_{n}^{\mathrm{HP}}\right]$$

### 4.8 Statistical Analysis

Statistical analyses were performed using GraphPad Prism version 10.0.2 (GraphPad Software, San Diego, CA, USA). Data points are expressed as mean ± standard deviation (s.d), unless otherwise indicated. As most datasets did not follow a normal distribution, non‐parametric tests were applied: The Mann–Whitney U test was used for comparisons between two groups, and the Kruskal–Wallis test was used for comparisons among three or more groups. Significance levels were denoted as follows: ^*^ *p* < 0.05, ^**^ *p* < 0.01, ^***^ *p* < 0.001, ^****^ *p* < 0.0001.

## Author Contributions

N.K.B., A.Z, F.Z., A.M.R., and S.Y. designed the research. S.Y., F.Z., I.G.C., A.M.R., and N.K.B. designed and/or carried experimental analyses. F.Z., S.Y., A.M.R, and A.Z. analyzed the motility data. F.Z., A.Z. and N.K.B. wrote and edited the manuscript. All authors approved the manuscript.

## Conflicts of Interest

The authors declare no conflicts of interest.

## Supporting information




**Supporting File**: smll72233‐sup‐0001‐SuppMat.docx

## Data Availability

The data that support the findings of this study are available from the corresponding author upon reasonable request.
