# Supplementary material for: Aggressive Glioblastoma Cells Enhance the Migratory Persistence and Velocity of Less Aggressive Cells to Promote Tumor Dissemination
Source: Small. 2026 Jan 8;22(11):e08142. doi: 10.1002/smll.202508142 (PMC12921552; doi:10.1002/smll.202508142)
Supplement: Supplementary file 1 — Supporting File: smll72233‐sup‐0001‐SuppMat.docx [file SMLL-22-e08142-s001.docx]

Supplementary file

**Aggressive glioblastoma cells enhance the migratory persistence and velocity of less aggressive cells to promote tumor dissemination**

*Fatima-ezzahra Ait Mohand^1^, Shaked Yemini^1^, Irina Gorobetz-Cojocari, Ariel M Rubinstein, Assaf Zemel*, Nataly Kravchenko-Balasha**

^1^ Equal contribution

* Corresponding authors

Nataly Kravchenko-Balasha [natalyk@ekmd.huji.ac.il](mailto:natalyk@ekmd.huji.ac.il)

Assaf Zemel [assafz@ekmd.huji.ac.il](mailto:assafz@ekmd.huji.ac.il)

The Institute of Biomedical and Oral Research, Hebrew University of Jerusalem, Jerusalem, 91120, Israel.

This file includes supplementary figures and supplementary methods.

**Supplementary Methods**

***Transfection with GFP and RFP***

To differentiate the GFP and RFP two subpopulations, we used the following transfection equation:

$Volume of {CellLight}^{®} Reagent (mL)=\frac{number of cells x desired PPC (particles per cell)}{1x{10}^{8} {CellLight}^{®} particles/mL}$.

The nuclei of the cells were also stained with Hoechst 33342 (SC-495790) at a concentration of 1:1000 (Hoechst:DMEM).

At least 16 hours after transfection, U87EGFRwt cells were seeded in a 24x well plate as a homogeneous culture or as a CC of either 10% U87EGFRvIII with 90% U87EGFRwt or 50% U87EGFRvIII with 50% U87EGFRwt, resulting in a final concentration of 3x10^4^ cells in each well. The cells were then incubated overnight (37°C, 5% CO2). The cells were imaged the next day on the Eclipse Ti Nikon microscope. Using a 10/0.3 Plan Fluor lens, multipoint snapshots of fields containing 100 cells/field were imaged at each time point. During the live imaging, the cells were kept in an incubation chamber, and a picture was taken every 10 minutes for 6 hours. NIS-Elements software was used to combine all the images into a video.

***Chip analysis***

2x10^4^ mcherry-labeled U87EGFRwt cells and 2x10^4^ unlabeled U87EGFRvIII cells were seeded onto the Arena A CYTOOchip^TM^ (CYTOO, #10-020-00-18) while it was mounted onto the 1-well CYTOOchamber (30-010). Each CYTOOchip Arena contains 2,704 adhesive round micropatterns with a diameter of 225 μm and separated by 100 μm, surrounded by a cytophobic surface that allows for the controlled attachment and spreading of cells only within the micropatterns. After seeding, the CYTOOchamber is placed in an incubator at 37°C and 5% CO2 for 6 hours. The cells are not incubated overnight to prevent cells from proliferating inside the micropatterns and increasing the number of cells constrained within the micropatterns. The medium is removed after incubation. To remove suspended cells that failed to adhere to the micropatterns during incubation, we washed the chip three times. The chip was then imaged on the Eclipse Ti Nikon microscope with the NIS-Elements program. Using a 10/0.3 Plan Fluor lens, multipoint snapshots of fields containing 16 micropatterns were imaged at each time point. During the live imaging, the cells were kept in an incubation chamber, and a picture was taken every 30 minutes for 6 hours.

***Laplace distributions of cell velocities***

Our data indicate that the components of cell velocity follow a Laplace distribution. The lines enveloping the histograms of the velocity components in Figure S5 are Laplace distributions given by:

|  | ${P(V}_{x})=P(V_{y})=\frac{\surd2}{2\sigma_{V_{x,y}}}\exp\left( -\surd2\left\vert V_{x,y} \right\vert/\sigma_{V_{x,y}} \right)$ | (1) |
| --- | --- | --- |
|  | P$\left( V_{p} \right)=\frac{\sqrt{2}}{2\sigma_{V_{p}}}\exp\left( -\surd2\left\vert V_{p}-\left\langle V_{p} \right\rangle\right\vert/\sigma_{V_{p}} \right), {P(V}_{n})=\frac{\surd2}{2\sigma_{V_{n}}}\exp\left( -\surd2\left\vert V_{n} \right\vert/\sigma_{V_{n}} \right)$ | (2) |

where $\sigma$ represents the respective standard deviation. The x and y components distribute identically by symmetry and their data was jointed to produce the histograms. Also, by symmetry, the *x*,*y* and *n* components have zero mean, but the mean velocity along the principal direction $\vec{p}$, $\left\langle V_{p} \right\rangle$, is non-zero and positive, indicating a bias to make a forward movements in the principal direction. From the Laplace distribution for the x,y components we have calculated the probability density function of the cellular speed, $\left| V \right|=\sqrt{V_{x}^{2}+V_{y}^{2}}$ with a suitable transformation of the random variables. Assuming $V_{x}$ and $V_{y}$ are independent Laplacian random variables one can show that the pdf of the cellular speed, $\left| V \right|$, is given by:

|  | P($\left\vert V \right\vert) =\frac{4\vert V\vert}{\sigma_{x,y}}\int_{0}^{\pi/4} exp[-\frac{\sqrt{2}\vert V\vert}{\sigma_{x,y}}\left[ cos\left( \theta\right)+sin\left( \theta\right) \right]]d\theta$ | (3) |
| --- | --- | --- |

The integral was calculated numerically to plot the enveloping solid lines in the speed distributions (panels D, H, L, P) of Fig. S4. From the expression above one can find the asymptotic behavior of the speed distribution for large $\left| V \right|$. For large $\left| V \right|$, the major contribution to the integral follows from the region where $\left[ cos\left( \theta\right)+sin\left( \theta\right) \right]$ is minimal, i.e., near $\theta$ =0. Replacing $\left[ cos\left( \theta\right)+sin\left( \theta\right) \right]$ by $1+\theta$ and integrating we find the following exponential asymptotic behavior at large $\left| V \right|$:

|  | P($\left\vert V \right\vert)\approx\frac{2\sqrt{2}}{\sigma_{x,y}}exp[-\frac{\sqrt{2}\vert V\vert}{\sigma_{x,y}}$] | (4) |
| --- | --- | --- |

Exponential distribution of cellular speeds has been found in previous studies^1,2^ and Wu et al^3^., has recently suggested that it may follow from the heterogeneity of cell motility characteristics in the population.

***Identification of highly persistent cells; cutoff optimization and validation:***

The approximating solid curves to the MSD data in Figure S5, were calculated from Eq. 9 using the appropriate persistence times extracted from the corresponding correlation functions (see methods section for explanation). The sorting of cells into a group of highly persistent (HP) cells and moderately persistent (MP) cells was made based on a cutoff level of cell persistence. Cells with $\tau_{p}^{i}\leq\tau_{p}^{\mathrm{cutoff}}=30 min$ were considered MP cells, and cells with $\tau_{p}^{i}>\tau_{p}^{\mathrm{cutoff}}$ were considered HP cells. Using higher values of$\tau_{p}^{\mathrm{cutoff}}$, shifted cells with higher persistence into the group of MP cells, consequently enhancing both $\tau_{p}^{\mathrm{MP}}$ and $\tau_{p}^{\mathrm{HP}}$, and vice versa. Based on the visual quality of the MSD fits and their quantitative $R^{2}$ measures (shown in Fig. S5A-E), we concluded that the range ${20 min<\tau}_{p}^{\mathrm{cutoff}}<40 min$ is most reliable for dissecting the small fraction of highly persistence (HP) cells form the population.

This was also consistent with the histogram of the single cell $\tau_{p}^{i}$ values shown in Fig. S5F, in which a mixture distribution, made of two log-normal distributions centered at $\left\langle\tau_{p}^{i} \right\rangle_{\mathrm{MP}}\mathrm{and}\left\langle\tau_{p}^{i} \right\rangle_{\mathrm{HP}}$, was fitted to the data. This figure too shows that the optimal range for separating the two groups of cells lies between 20-40 min. In addition, the in-silico analysis discussed in the next section of mixed MP/HP populations revealed the same optimal range for distinguishing the two groups of cells. Using $\tau_{p}^{\mathrm{cutoff}}$=30 min we obtained best $R^{2}$ scores to the fitting of our MSD data we hence used this value for analyzing our data.

It should be noted, however, that due to the small number of HP cells in culture (20 - 30 cells) the value we obtain for $\tau_{p}^{\mathrm{HP}}$ is sensitive to our choice of $\tau_{p}^{\mathrm{cutoff}}$ (e.g., for EGFRwt, we find $\tau_{p}^{\mathrm{HP}}=65 \pm10$for $\tau_{p}^{\mathrm{cutoff}}$=20 and $\tau_{p}^{\mathrm{HP}}=85 \pm15$ for $\tau_{p}^{\mathrm{cutoff}}$=40 and for EGFRvIII, we find $\tau_{p}^{\mathrm{HP}}=90\pm20$ min for $\tau_{p}^{\mathrm{cutoff}}$=20 and $\tau_{p}^{\mathrm{HP}}=290 \pm30$ for $\tau_{p}^{\mathrm{cutoff}}$=40). Nevertheless, the $\tau_{p}^{\mathrm{HP}}$value calculated for a given $\tau_{p}^{\mathrm{cutoff}}$, was well suited to compare between conditions, e.g., between that of EGFRwt and EGFRvIII cells and between all different cc conditions. Importantly, we have verified that all the preformed statistical-significance tests and the conclusions drawn from them were unaltered upon varying the $\tau_{p}^{\mathrm{cutoff}}$ value within the 20-40 min interval. Finally, unlike for the HP cells, for the majority of MP cells we find, $\tau_{p}^{\mathrm{MP}}\approx5-8$ min under all conditions consistent with previous reports^4^.

***In silico cell trajectories from simulations of anisotropic Ornstein-Uhlenbeck processes***

To support the analysis of our experimental cell trajectories and the identification of distinct MP and HP cell subpopulations, we performed similar analysis with *in-silico* generated ensembles of cell trajectories. To this end we ran computer simulations of "cellular" particles who's instantaneous $x$ and $y$ coordinates have been derived from independent 1D Ornstein-Uhlenbeck (OU) processes fulfilling the following stochastic differential equations:

|  | $\frac{dV_{x}}{dt}=-\frac{1}{\tau_{x}}V_{x}+\gamma_{x} \xi\left( t \right), \frac{dV_{y}}{dt}=-\frac{1}{\tau_{y}}V_{y}+\gamma_{y} \xi\left( t \right)$ | (1) |
| --- | --- | --- |

The OU model is a common (minimal) model of cell motility^2,3,5^ and of a persistent random walk more generally. It accounts for two basic elements of cell motility. The first, modeled with the second term on the right, is a random force accounting for instantaneous random changes in cell velocity. It is modeled by an uncorrelated Gaussian white noise, $\xi\left( t \right)$, with corresponding magnitudes $\gamma_{x},$ $\gamma_{y}$ in the two directions. The second contribution, included with the first term in Eq. 1 represents a drag force which accounts for viscous and frictional interactions both within the cell and between the cell and the substrate. This force dampens cell acceleration caused by the random force and restores the cell velocity to its stationary mean level over the time scales $\tau_{x}$​ and $\tau_{y}$. The magnitudes of the random force, $\gamma_{x},$ $\gamma_{y}$ are related to the stationary mean square velocity components via, $2\left\langle V_{x}^{2} \right\rangle=\tau_{x}\gamma_{x}^{2}$ and $2\left\langle V_{y}^{2} \right\rangle=\tau_{y} \gamma_{y}^{2}$, and we also have $\left\langle V_{x} \right\rangle=\left\langle V_{y} \right\rangle=0$ by symmetry. In addition, the stationary velocity autocorrelation function for the above anisotropic OU process is given by:

| $C_{\vec{V}}\left( t \right)=\left\langle\vec{V}\left( t+t_{0} \right)\circ\vec{V}\left( t_{0} \right) \right\rangle=\left\langle V_{x}\left( t+t_{0} \right)V_{x}\left( t_{0} \right) \right\rangle+\left\langle V_{y}\left( t+t_{0} \right)V_{y}\left( t_{0} \right) \right\rangle=\left\langle V_{x}^{2} \right\rangle\exp\left( -t/\tau_{x} \right)+\left\langle V_{y}^{2} \right\rangle\exp\left( -t/\tau_{y} \right)$ | (2) |
| --- | --- |

The two components $C_{x}\left( t \right)=\left\langle V_{x}\left( t+t_{0} \right)V_{x}\left( t_{0} \right) \right\rangle$ and $C_{y}\left( t \right)=\left\langle V_{y}\left( t+t_{0} \right)V_{y}\left( t_{0} \right) \right\rangle$ decay exponentially with the respective persistence-times, $\tau_{x}$ and $\tau_{y}$. Furthermore, with the above velocity autocorrelation function one finds the following expression for the mean square displacement$:$

| $\left\langle\vec{R}^{2}\left( t \right) \right\rangle=2\left[ \tau_{x} \left\langle V_{x}^{2} \right\rangle\left[ t-\tau_{x} \left[ 1 - \exp\left( -t/\tau_{x} \right) \right] \right]+\tau_{y} \left\langle V_{y}^{2} \right\rangle\left[ t-\tau_{y} \left[ 1 - \exp\left( -t/\tau_{y} \right) \right] \right] \right]$ | (3) |
| --- | --- |

The mean square velocity components, $\left\langle V_{x}^{2} \right\rangle$, $\left\langle V_{y}^{2} \right\rangle$ and the two persistence times, $\tau_{x}$, $\tau_{y}$ were used as input parameters for simulating *in*-*silico* cell trajectories. The simulations were performed using the built-in function, $\mathrm{OrnsteinUhlenbeckProcess}[ ]$, of the Wolfram Mathematica software, using a time step, $dt=10$ min, to mimic the time step of image acquisition in our experimental cell data. Using this function, we created ensembles of cell trajectories with defined values of $\left\langle V_{x}^{2} \right\rangle$, $\left\langle V_{y}^{2} \right\rangle$ and $\tau_{x}$, $\tau_{y}$. To mimic our experimental cell data, we generated trajectories that were anisotropic both in the mean square velocity and the persistence-time. Using $\left\langle V_{x}^{2} \right\rangle$> $\left\langle V_{y}^{2} \right\rangle$ and $\tau_{x}$ > $\tau_{x}$, we obtained random trajectories with a mean spatial bias along the $x$-direction. We then carried out the same analysis as done with our experimental cell trajectories. To each cell trajectory we first calculated a principal direction $\vec{p}$ and a normal direction $\vec{n}$; due to the finite simulation duration, each of these slightly and randomly deviated from the expected mean directions $\left\langle\vec{p} \right\rangle=\hat{x}$ and $\left\langle\vec{n} \right\rangle=\hat{y}$. We then calculated, for each trajectory, i, the corresponding two components of thevelocity autocorrelation function $C_{p}^{i}\left( t \right)=\left\langle V_{p}^{i}\left( t+t_{0} \right)V_{p}^{i}\left( t_{0} \right) \right\rangle_{t_{0}}$ and $C_{n}^{i}\left( t \right)=\left\langle V_{n}^{i}\left( t+t_{0} \right)V_{n}^{i}\left( t_{0} \right) \right\rangle_{t_{0}}$ which sum to the overall autocorrelation function of that cell trajectory,

$C_{\vec{V}}^{i}\left( t \right)=\left\langle\vec{V}^{i}\left( t+t_{0} \right)\circ\vec{V}^{i}\left( t_{0} \right) \right\rangle_{t_{0}}=C_{p}^{i}\left( t \right)+C_{n}^{i}\left( t \right)$,

and extracted the respective single-cell persistence-times $\tau_{p}^{i}$ and $\tau_{n}^{i}$ by fitting the functions to decaying exponentials. In the equations above, averaging was performed over the possible starting points, ($t_{0}$), within a trajectory using the overlapping interval method^6^ . In addition to these single-cell characteristics, we also calculated the *ensemble-averaged* correlation functions, $C_{p}\left( t \right)=\frac{1}{N}\sum_{i=1}^{N} C_{p}^{i}\left( t \right)$ and $C_{n}\left( t \right)=\frac{1}{N}\sum_{i=1}^{N} C_{n}^{i}\left( t \right)$, which sum to the overall correlation function, $C_{\vec{V}}\left( t \right)=C_{p}\left( t \right)+C_{n}\left( t \right)$, and extracted the characteristic persistence times, $\tau_{p}$ and $\tau_{n}$ of the entire ensemble, again by fitting these functions with decaying exponentials. We note that these values are generally smaller than or equal to the ensemble-averaged single-cell persistent times, namely, $\tau_{p}\leq\left\langle\tau_{p}^{i} \right\rangle_{N}$= $\frac{1}{N}\sum_{i=1}^{N} \tau_{p}^{i}$, and $\tau_{n}\leq\left\langle\tau_{n}^{i} \right\rangle_{N}$= $\frac{1}{N}\sum_{i=1}^{N} \tau_{n}^{i}$, and are more accurate characteristics of the ensemble since the extraction of single-cell persistence times comes with its own fitting error. For sufficiently long simulations, T$\gg\tau_{p},\tau_{n}$, one expects all the single-cell $\tau_{p}^{i}$, $\tau_{n}^{i}$ values and the ensemble averaged $\tau_{p}$, $\tau_{n}$ values to converge to the original input values $\tau_{p}^{\mathrm{inp}}=\tau_{x}$, ${\tau_{n}^{\mathrm{inp}}=\tau}_{y}$. However, the use of finite T is associated with an inherent methodological variability in the extracted persistence times. The same situation also occurs with our experimental data but the experiments, of course, are more complex since they encompass additional biological sources of variability. We thus used our simulations to quantify the methodological variability associated with the use of a finite simulation length, T$=350 \min\approx5 - 40 \tau_{p}$, and finite time step, $dt=10 \min$, as used in our experimental data acquisition. The calculation of single-cell persistence times and their variability was done for identifying distinct subpopulations which differed by their persistence. To this end we first characterized the distributions resulting with homogeneous populations where a single set of input parameters, $\left\langle V_{p}^{2} \right\rangle_{\mathrm{inp}}$, $\left\langle V_{n}^{2} \right\rangle_{\mathrm{inp}}$ and $\tau_{p}^{\mathrm{inp}}$, $\tau_{n}^{\mathrm{inp}}$ was used, and then examined a mixture of two subpopulations as found in our experiments.

The results of this analysis are summarized in Figure S6. For concreteness we used the values extracted for the WT. As a model for the moderately persistent (MP) group, we took: $\tau_{p}^{\mathrm{inp}}=8$ min, and $\tau_{n}^{\mathrm{inp}}=5$ min, and for the highly persistent (HP) group we took: $\tau_{p}^{\mathrm{inp}}=70$ min and $\tau_{n}^{\mathrm{inp}}=5$ min; the mean square velocity components were, $\left\langle V_{p}^{2} \right\rangle_{\mathrm{inp}}= 0.7 ({\mu m/min)}^{2}$, $\left\langle V_{n}^{2} \right\rangle_{\mathrm{inp}}= 0.3 ({\mu m/min)}^{2}$ in both cases. Panels A and B, respectively, show the results for the two subpopulations separately, and panel C is for their mixture. To obtain clearer statistics we used 300 cells for each group. Panels A1, A2 and B1, B2 show histograms of $\tau_{p}^{i}$ and $\tau_{n}^{i}$ values, the overall simulation time was T=350 min as in our experiments. We found the histograms to be well fit by a log-normal distribution (black dashed curve), they peaked around the corresponding input value and their standard deviation was proportional to the input persistence time. The relatively wide tail of the distributions is a consequence of the finite simulation length. Running 10-fold longer simulations with T=3500 min (panels A3, A5, B3, B4) resulted in significantly sharper distributions. The measurements of single-cell $\tau_{p}^{i}$ or $\tau_{n}^{i}$ values that are several-fold higher than the input value in shorter simulations merely reflect the characterization of rare episodes in an otherwise longer trajectory. This is clear from the two example trajectories in panels A2 and A4 which were calculated with $\tau_{p}^{\mathrm{inp}}=8$min for T=350 min and T=3500 min, and for which we found $\tau_{p}^{i}=66 \pm13$ min and $\tau_{p}^{i}=9.3\pm1.6$ min, respectively. While the cell in panel A2 appears to be moving quite persistently for the entire trajectory, the blue-purple segment of the trajectory seen in panel A4 shows that this might be a non-characteristic episode in a longer trajectory.

We next simulated a mixture of the two cell subpopulations comprising 100 MP cells with $\tau_{p}^{inp}=8$ min and $\tau_{n}^{inp}=5$ min and 20 HP cells with $\tau_{p}^{inp}=70$ min and $\tau_{n}^{inp}=5$ min. Panel C1 shows the resulting distribution of persistence times, $\tau_{p}^{i}$. The enveloping black curve is a fit to mixture distribution with two log-normal components one peaked at $\tau_{p}^{i}=8$ min and the other at $\tau_{p}^{i}=70$ min. Prominent in this analysis is the shallowness of the HP cells contribution resulting firstly due to the minority (16%) of this group and secondly because of the large standard deviation, $\sigma_{\tau_{p}^{i}}\approx\tau_{p}^{inp}=70$ min, of that subpopulation. This makes it complicated to identify the HP subpopulation within this mixture. However, the $\tau_{p}^{i}$ distribution of the MP group is panel A shows the extent of the methodological variability in our calculations. From the cumulative distribution function (CDF) shown in the inset figure in panel A1 one can deduce that with probability of 98% a measured $\tau_{p}^{i}$ will be smaller than a cutoff value of say $\tau_{p}^{\mathrm{cutoff}}=30$ min; this is shown with the red vertical line that marks the position of the cutoff value. One can obviously choose a higher value to account for all the MP cells, but the probability that a cell with $\tau_{p}^{i}>30$ falls within the domain of the HP group increases, we calculate that it will with a probability of 70%. Returning to panel C1 we conclude that using a cutoff level in the range 20-30 min is reasonable for identifying the two subpopulations in our ensemble. These considerations support our analysis of our experimental data. The calculated theoretical distribution of panel C1 appears similar to the one found for the WT and shown in panel D1 revealing that the variability arising from the finite duration of the cell trajectories is a major component of the persistence time variability and that the choice of $\tau_{p}^{\mathrm{cutoff}}\approx30$ min is appropriate for sorting out an HP subtype from the ensemble.

Furthermore, the existence of distinct subpopulations that differ in their persistence time is also apparent in the ensembles' velocity autocorrelation function shown in panel C2 for OU ensemble and in panel D2 for the WT. Both clearly show the cross-over between the two timescales. This should be compared to the one shown in panel B5 that was calculated for the homogeneous ensemble calculated with $\tau_{n}^{inp}=5$ min and $\tau_{p}^{inp}=70$ min. The crossover between the two intrinsic time scales (see the black arrow) is mild since the contribution of the more rapidly fluctuating normal component, $C_{n}\left( t \right),$ to the total velocity correlation function is only 30% as dictated by the fraction $C_{n}\left( 0 \right)/C_{\vec{V}}\left( 0 \right) =\left\langle V_{n}^{2} \right\rangle$/($\left\langle V_{n}^{2} \right\rangle+\left\langle V_{p}^{2} \right\rangle=0.3.$

Finally, we note that the values we extract for the persistence times of the HP group may not be accurate representatives of that group. This is due both to the small number of cells identified as HP cells and the relatively short duration of the experiment that extends only a few persistence-times, $T/\tau_{p}=3-5$. Our simulations reveal that the error in estimating the original input value of $\tau_{p}$ is 5% for the MP group and 30% for the HP group. Thus while the standard errors we quate for the extracted values of $\tau_{p}$ based on the quality of exponential fits to the autocorrelation function are much smaller, the values may still not be accurate typifies of one unique subpopulation. Moreover, our data is too limited to tell if what we identify as HP via the use of a cutoff comprise one or more subgroups.

**
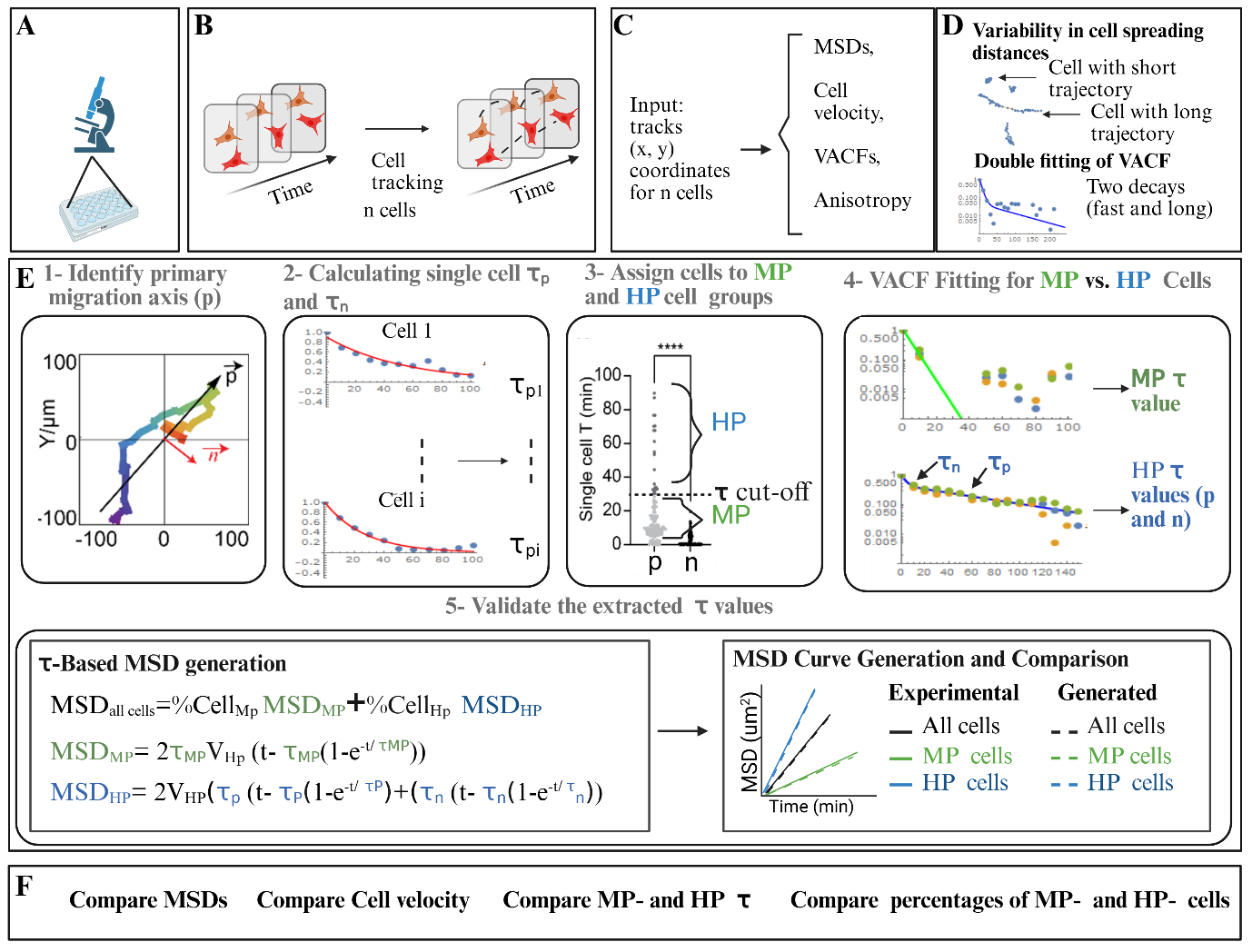
Supplementary figures (S1-S8)**

**Figure S1:** **Workflow of the experimental - theoretical analysis**.

**(A)** Time-lapse microscopy. 24 hours after seeding, treatment was applied, and cells were imaged for a duration of 6 hours, with images captured every 10 or 30 minutes. **(B)** Data collection involves tracking the positions of cells (x, y coordinates) at multiple time points. **(C)** Data processing step includes the calculation of MSDs (Mean Squared Displacement), cell velocity, VACFs (Velocity Autocorrelation Function) for each tracked cell.
**(D)** Insights-driven analysis shows variability in cell spreading distances and double fitting of VACF (fast and slow decays). **(E)** Post-processing analysis: (1) Identification of the primary direction migration; (2) Single-cell τ_p_ and τ_n_ values are calculated for each cell; (3) Cells are assigned to either moderately persistent (MP) or highly persistent (HP) cell categories; (4) VACF fitting for moderate and high τ (primary (P) and non-primary (N) axis) cells; (5) Validation of the extracted τ values is performed by comparing the theory-based (generated) MSD curves and experimental MSD plots. **(F)** Comparative analysis of cell spreading across co-cultures and treatments, comparing MSDs, cell velocity between MP and HP cells.

**
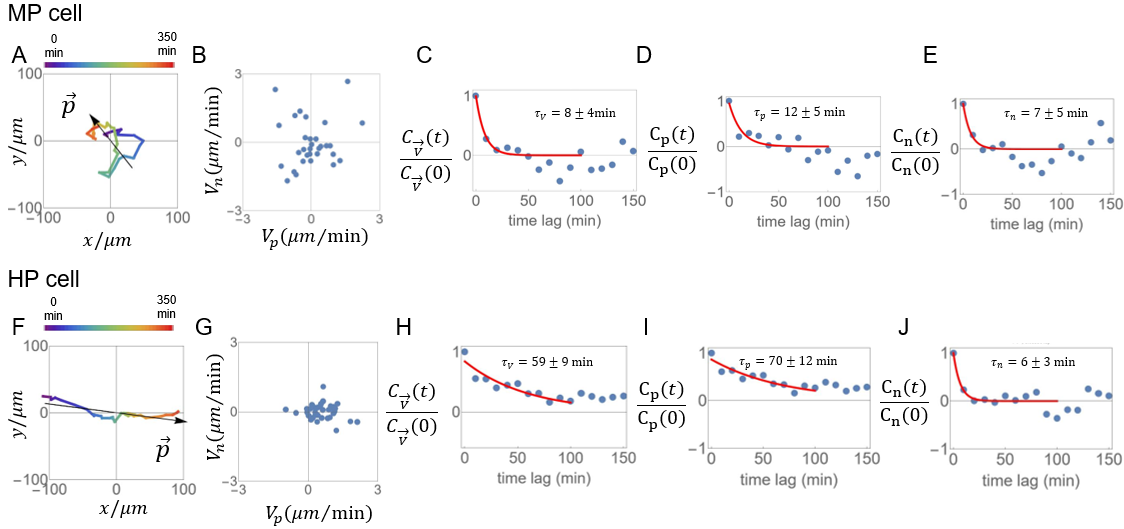
**

**Figure S2: Analysis of representative single cell trajectories: (A-E)** a moderately persistent (MP) cell**,** and **(F-J)** a highly persistent (HP) cell; both of WT subtype. (**A, F)** show the $x,y$ coordinates along the cells' trajectories with blue to red colors indicating the course of movement and the arrow indicating the calculated trajectory's principal direction, $\vec{p}$**. (B, G)** are scatter plots of the velocity components (${V_{p},V}_{n})$along the trajectories; note the velocity anisotropy in the HP cell trajectory (panel G). Panels **C-E** and **H-J** show the respective velocity autocorrelation functions. (**C, H**) the (scaled) total velocity, autocorrelation function**,** ${C_{\vec{V}}(t)}/{C_{\vec{V}}(0)}$. (**D, I**) the (scaled) velocity's p-component autocorrelation function, ${C_{p}(t)}/{C_{p}(0)}$, and (**E, J**) the (scaled) velocity's n-component autocorrelation function, ${C_{n}(t)}/{C_{n}(0)}$. Red curves are fitted exponentials, with the corresponding extracted persistence-times written above the plots. Note in the HP cell example the 10-fold higher persistence-time along the $\vec{p}$ direction in comparison to the $\vec{n}$ direction.

**
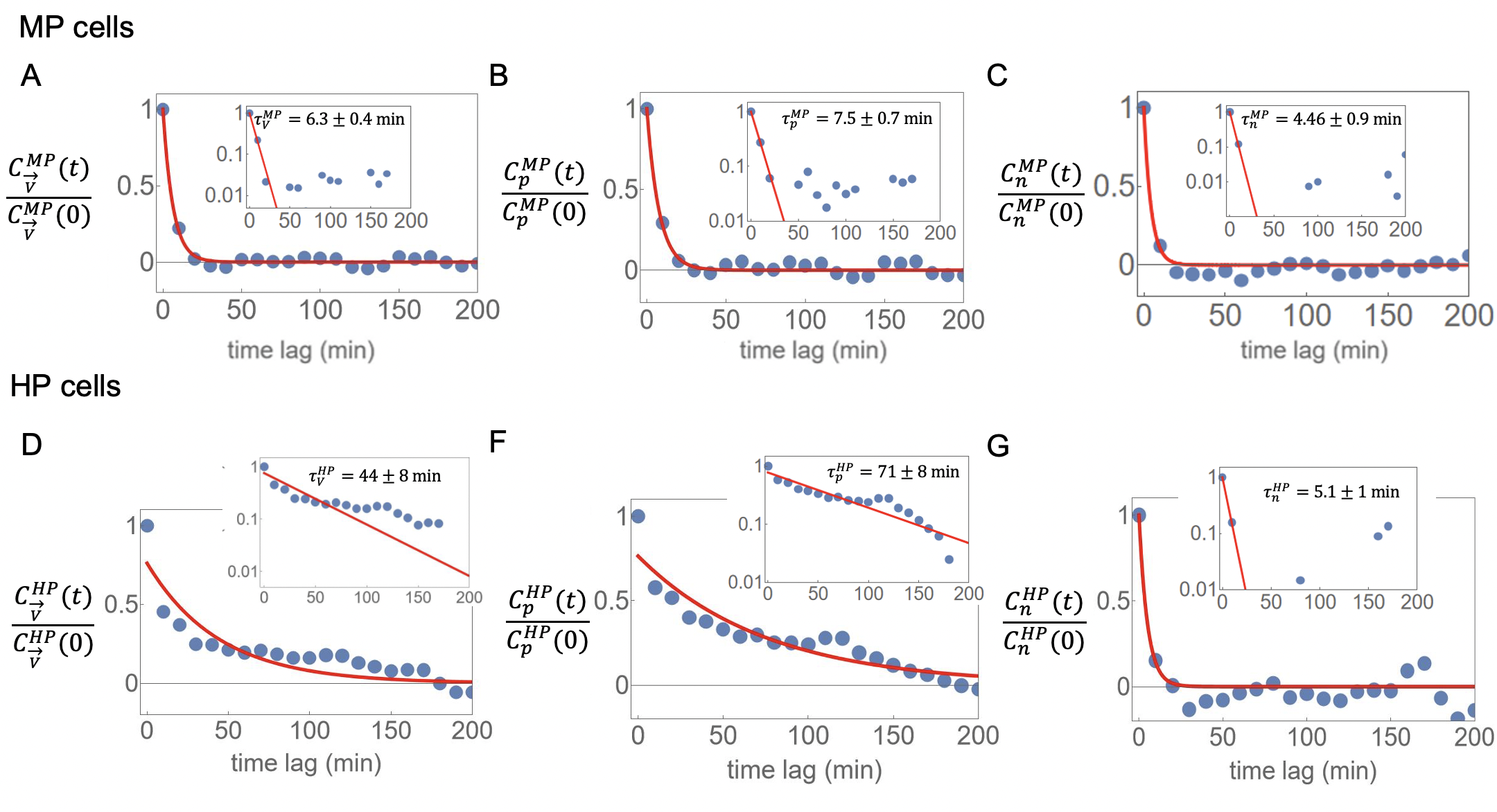
**

**Figure S3: Velocity autocorrelation functions and the extracted persistence-times of EGFRwt cell trajectories: (A-C)** correspond to the moderately persistent (MP) subpopulation of cells**,** and (**D-F)** to the highly persistent (HP) subpopulation. (**A, D**) show the (scaled) total velocity autocorrelation function**,** ${C_{\vec{V}}(t)}/{C_{\vec{V}}(0)}$. (**B, E**) the (scaled) velocity's p-component autocorrelation function, ${C_{p}(t)}/{C_{p}(0)}$, and (**C, F**) the (scaled) velocity's component autocorrelation function, ${C_{n}(t)}/{C_{n}(0)}$. Red curves are fitted exponentials, $a \exp\left( -t/\tau\right)$, with the corresponding extracted persistence-times, $\tau$, written above the plots. Note the 10-fold higher persistence-time along the $\vec{p}$ direction (compared to the $\vec{n}$ direction) in the HP group of cells.

**
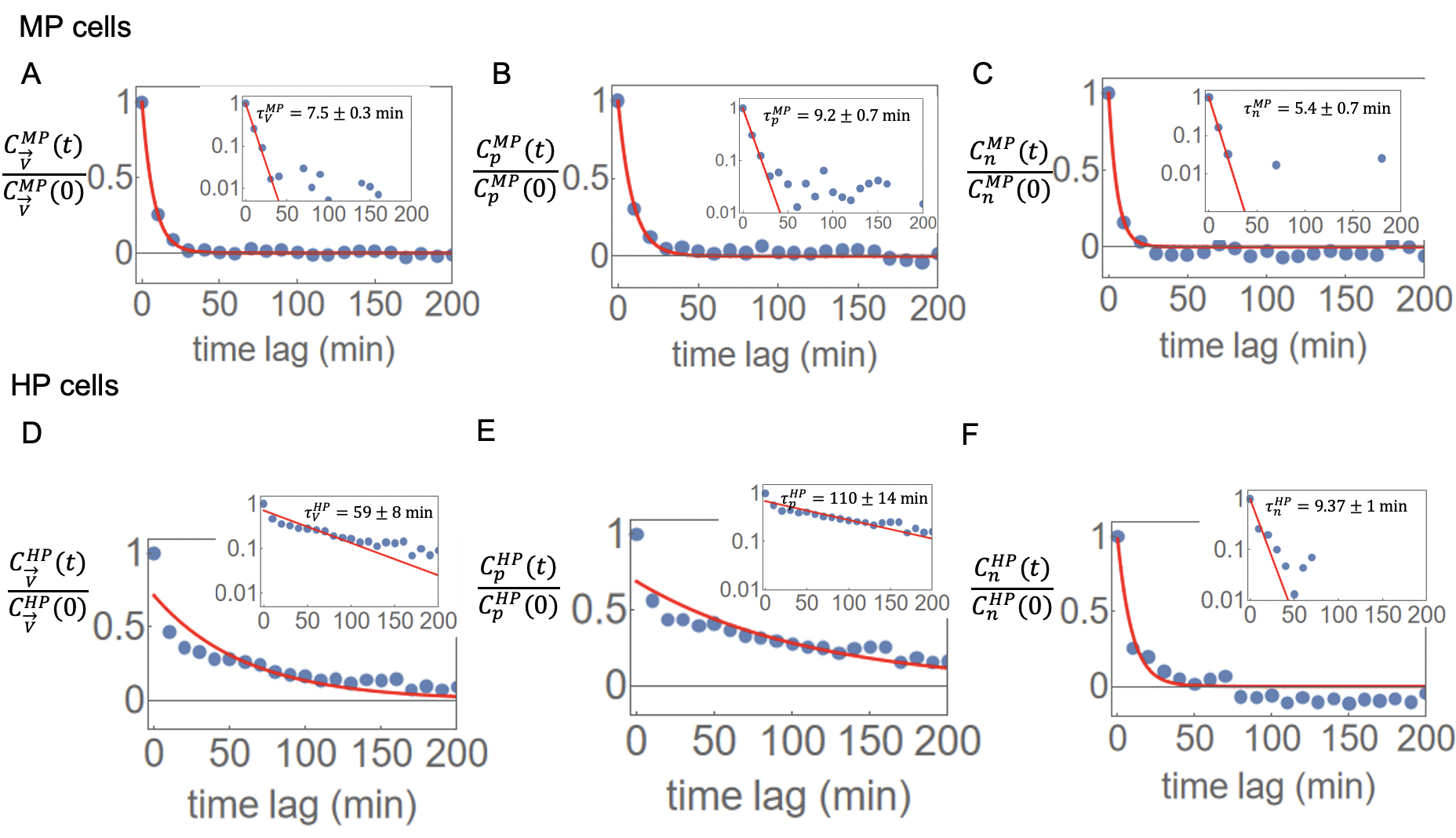
**

**Figure S4: Velocity autocorrelation functions and corresponding persistence-times of the EGFRvIII cell trajectories: (A-C)** correspond to the moderately persistent (MP) subpopulation of cells**,** and **(D-F)** correspond to the highly persistent (HP) subpopulation. (**A, D**) show the (scaled) total velocity autocorrelation function**,** ${C_{\vec{V}}(t)}/{C_{\vec{V}}(0)}$(**B, E**) the (scaled) velocity's p-component autocorrelation function, ${C_{p}(\delta t)}/{C_{p}(0)}$, and (**C, F**) the (scaled) velocity's n-component autocorrelation function, ${C_{n}(t)}/{C_{n}(0)}$. Red curves are fitted exponentials, $a \exp\left( -t/\tau\right)$, with the corresponding extracted persistence-times, $\tau$, written above the plots. Note the 10-fold higher persistence-time along the $\vec{p}$ direction (compared to the $\vec{n}$ direction) in the HP group of cells.


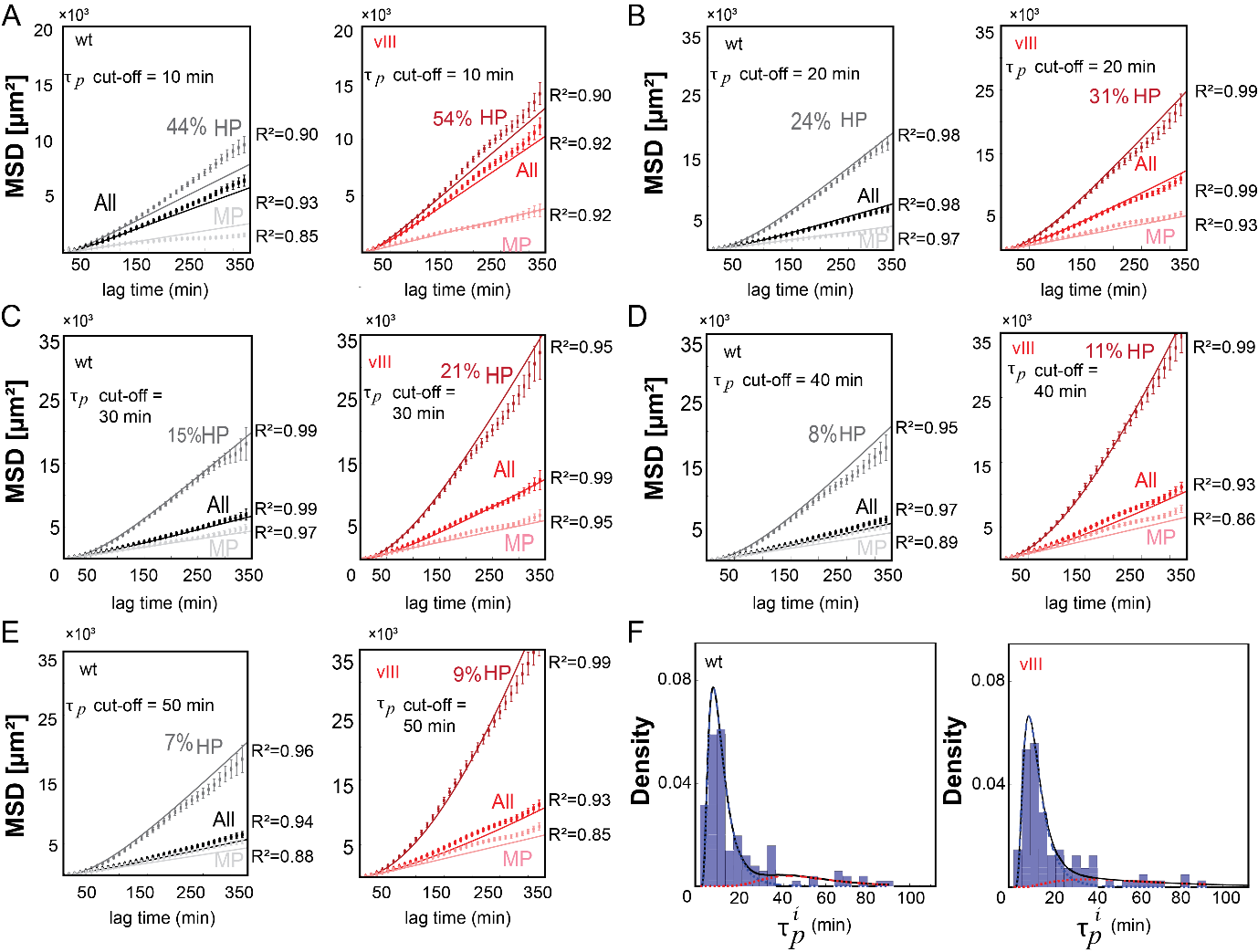


**Figure S5: Validation of τₚ cutoff selection using MSD fitting performance EGFRwt and EGFRvIII cells.**

**A-E**: Experimental MSD data and their corresponding theoretical MSD fits (solid lines) as calculated from Eq. 9, for EGFRwt (left) and EGFRvIII (right) populations using five different cutoff values, $\tau_{p}^{\mathrm{cutoff}}$=10, 20, 30, 40, and 50 minutes. For each cutoff, MSD curves are shown for the highly persistent (HP) subgroup, the moderately persistent (MP) subgroup, and the entire population (All). The percentage of trajectories classified as HP cells under each cutoff is indicated in each panel. R² scores were calculated using data only up to 250 minutes due to the inherent larger statistical error in the evaluation of the MSD data beyond this point. **F**: Distributions of single cell persistence times, τ^i^ₚ, for EGFRwt and EGFRvIII cells. The data were fitted with a mixture distribution comprised by two log-normal distributions centered at $\left\langle\tau_{p}^{i} \right\rangle_{\mathrm{MP}}\mathrm{and}\left\langle\tau_{p}^{i} \right\rangle_{\mathrm{HP}}$; the **blue curve** represents the MP component, the **red curve** represents the HP component, and the **black curve** shows the combined mixture fit. This analysis highlights the long-tailed nature of the distribution of the single cell persistence times and indicates that the range between 20–40 min is most suitable for delineating the HP group from the population.

**
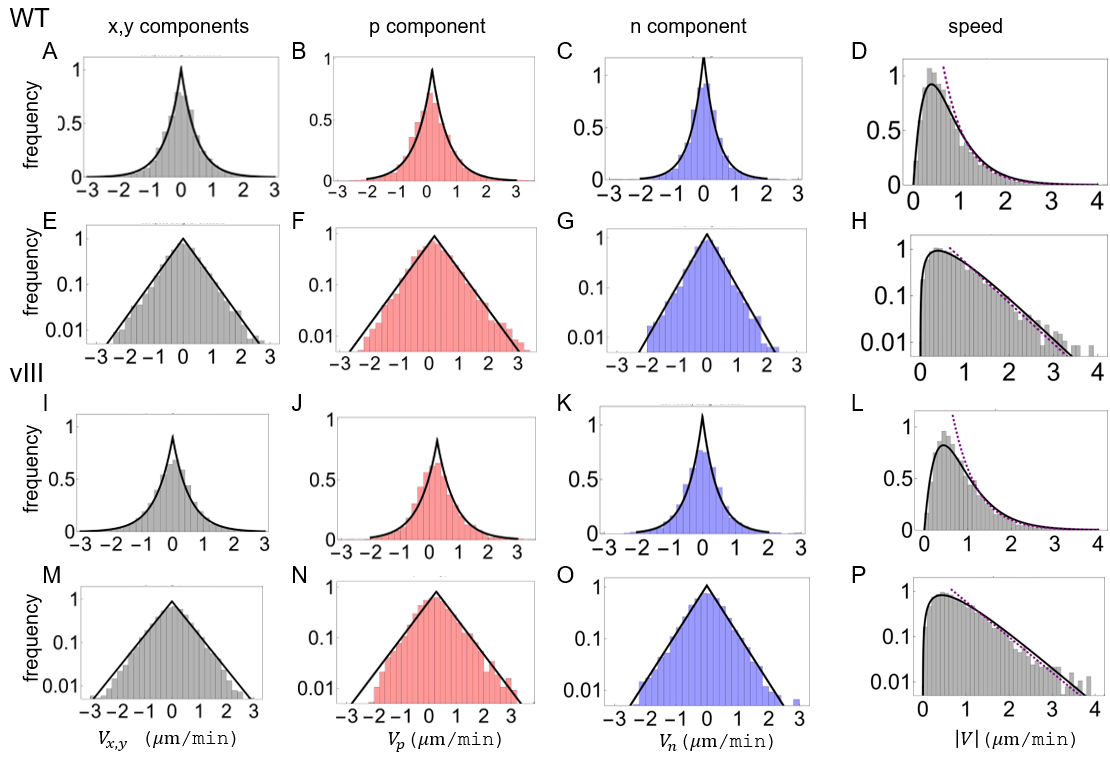
**

**Figure S6: Velocity distributions of EGFRwt and EGFRvIII cell trajectories: Panels A-H correspond to EGFRwt cells and I-P to EGFRvIII cells.** Three first columns from left to right respectively show the distribution of the x,y-components, p-component, and n-component of cell velocity, and the left most column is the magnitude of cell velocity. A-D and I-L show the distributions in linear scale. E-H and M-P show the distributions in logarithmic scale. The solid black curves enveloping the distributions of the velocity components are Laplace distributions calculated with the mean and variance of the respective distributions. The solid black lines enveloping the speed distributions (panels D, H, L, P) were calculated from the Laplace distributions of the x,y components, V_(x,) and V_y with a suitable transformation of variables. The dashed purple curves in panels D, H, L, P are exponential asymptotes to the speed distributions.

***
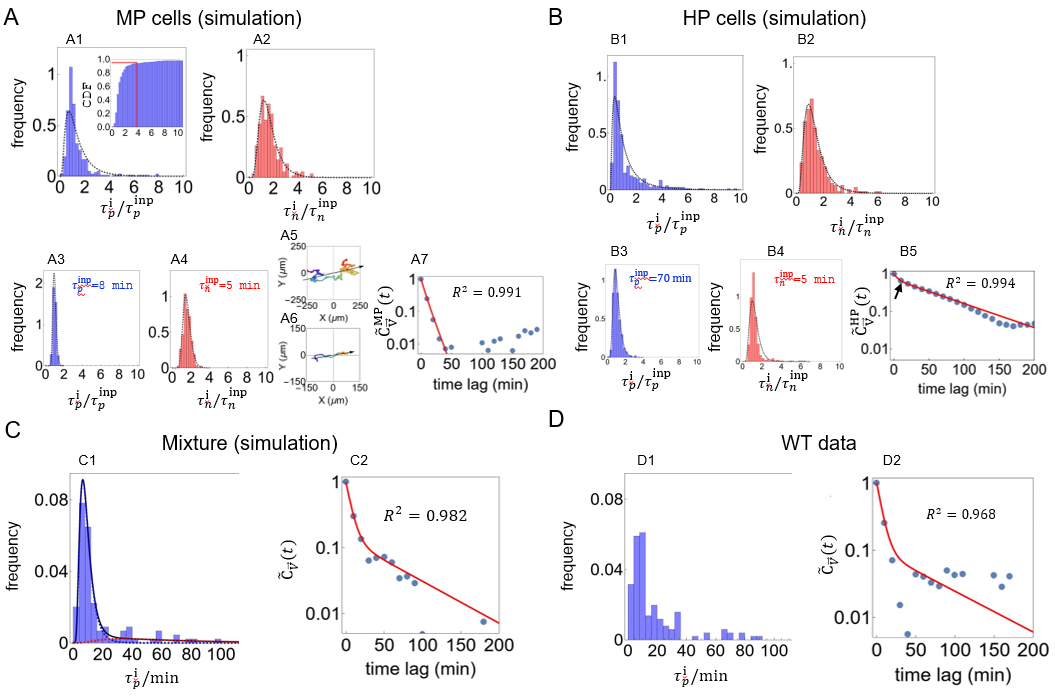
***

|  |
| --- |
|  |

**Figure S7: Analysis of simulation data for ensembles of (A) moderately persistent (MP) cells, (B) highly persistent (HP) cells and (C) a mixture of the two, compared with EGFRwt (100%wt) data in (D).**

Panels **A1, A2** and **B1, B2** show histograms of single-cell persistence times along the $\vec{p},\vec{n}$ directions for the MP/HP groups, respectively. As input values, we used the persistence times and mean square velocities obtained for the WT subtype. The histograms peak at the corresponding input value $\tau_{p}^{i}\approx\tau_{p}^{\mathrm{inp}}$ and $\tau_{n}^{i}\approx\tau_{n}^{inp}$ and their standard deviation (widths) scale with these values, $\sigma_{\tau_{p}^{i}}\sim$ $\tau_{p}^{\mathrm{inp}}$ and $\sigma_{\tau_{n}^{i}}\sim$ $\tau_{n}^{\mathrm{inp}}$. The histograms are well fit with a log-normal distribution (dashed curves) and their relatively wide tail is a consequence of the finite overall simulation duration, T=350 min. Indeed, with 10-fold longer simulations (T=3500 min) we obtained considerably sharper distributions as seen in panels **A3, A4** and **B3, B4**. The **inset in panel A1** is the cumulative distribution function (CDF) with the red line marking the position of the cutoff value $\tau_{p}^{\mathrm{cutoff}}$=30 min used for sorting the trajectories into MP and HP groups. The example trajectories in **A5** and **A6**, calculated for T=3500 min and T=350 min, respectively, with the same input values $\tau_{p}^{\mathrm{inp}}$=8 min $\tau_{n}^{\mathrm{inp}}$=5 min, reveal how a relatively long persistence time, $\tau_{p}^{i}$=66 min, can be extracted in a short simulation. **A7, B5** show the *ensemble-averaged* (scaled) velocity autocorrelation functions $\tilde{C}_{\vec{V}}^{\mathrm{MP}}\left( t \right)=C_{\vec{V}}^{\mathrm{MP}}\left( t \right)/C_{\vec{V}}^{\mathrm{MP}}\left( 0 \right)$ and $\tilde{C}_{\vec{V}}^{\mathrm{HP}}\left( t \right){=C}_{\vec{V}}^{\mathrm{HP}}\left( t \right)/C_{\vec{V}}^{\mathrm{HP}}\left( 0 \right)$ for the MP and HP groups, respectively. For these two groups we obtained $\tau_{p}^{MP}=8.6\pm0.3$ min, $\tau_{n}^{MP}=4.7\pm0.9$ min and $\tau_{p}^{HP}=67\pm1$ min, $\tau_{n}^{HP}=5.6\pm0.6$ min. The red curves are plots of the double exponent $\left\langle V_{p}^{2} \right\rangle_{MP}/[\left\langle V_{p}^{2} \right\rangle_{MP}+\left\langle V_{n}^{2} \right\rangle_{MP}] exp\left( -t/\tau_{p}^{MP} \right)+\left\langle V_{n}^{2} \right\rangle_{MP}/[\left\langle V_{p}^{2} \right\rangle_{MP}+\left\langle V_{n}^{2} \right\rangle_{MP}]exp\left( -t/\tau_{n}^{MP} \right)\approx C_{\vec{V}}^{\mathrm{MP}}\left( t \right)/C_{\vec{V}}^{\mathrm{MP}}\left( 0 \right)$, and a corresponding function for the HP group. For the MP group we obtained a good fit with a single exponent with $\tau_{\vec{V}}^{MP}=7.1\pm0.1$ min. Panel **C** shows simulation data for a mixture of 100 MP and 20 HP cell trajectories generated using the input values used in panels (A) and (B). **C1** is the $\tau_{p}^{i}$ distribution and **C2** the scaled velocity autocorrelation function $\tilde{C}_{\vec{v}}\left( t \right)$. The red curve is a plot of the approximating triple-exponent ${(20/120)[\left\langle V^{2} \right\rangle}_{MP}\exp\left( -t/\tau_{\vec{V}}^{MP} \right)]+(100/120)[\left\langle V_{n}^{2} \right\rangle_{MP}\exp\left( -t/\tau_{n}^{HP} \right)+\left\langle V_{p}^{2} \right\rangle_{MP}\exp\left( -t/\tau_{p}^{HP} \right)]\approx C_{\vec{V}}\left( t \right)/C_{\vec{V}}\left( 0 \right)$, with the values $\tau_{\vec{V}}^{MP}=6.8\pm0.3$ min , $\tau_{n}^{HP}=4\pm1$ min and $\tau_{p}^{HP}=68\pm4 min$, obtained by fitting the respective correlation functions $C_{\vec{V}}^{\mathrm{MP}}\left( t \right)$, $C_{n}^{\mathrm{HP}}\left( t \right)$ and $C_{p}^{\mathrm{HP}}\left( t \right)$ with single exponents. For comparison, in panel **D** we provide the corresponding WT data, for which we found, $\tau_{n}^{MP}=4.4\pm0.9$ min, $\tau_{p}^{MP}=7.5\pm0.7$min, $C_{\vec{V}}^{\mathrm{MP}}\left( t \right)=6.3\pm0.4$ and $\tau_{n}^{HP}=5\pm1$ min and $\tau_{p}^{HP}=71\pm8 min$.

**
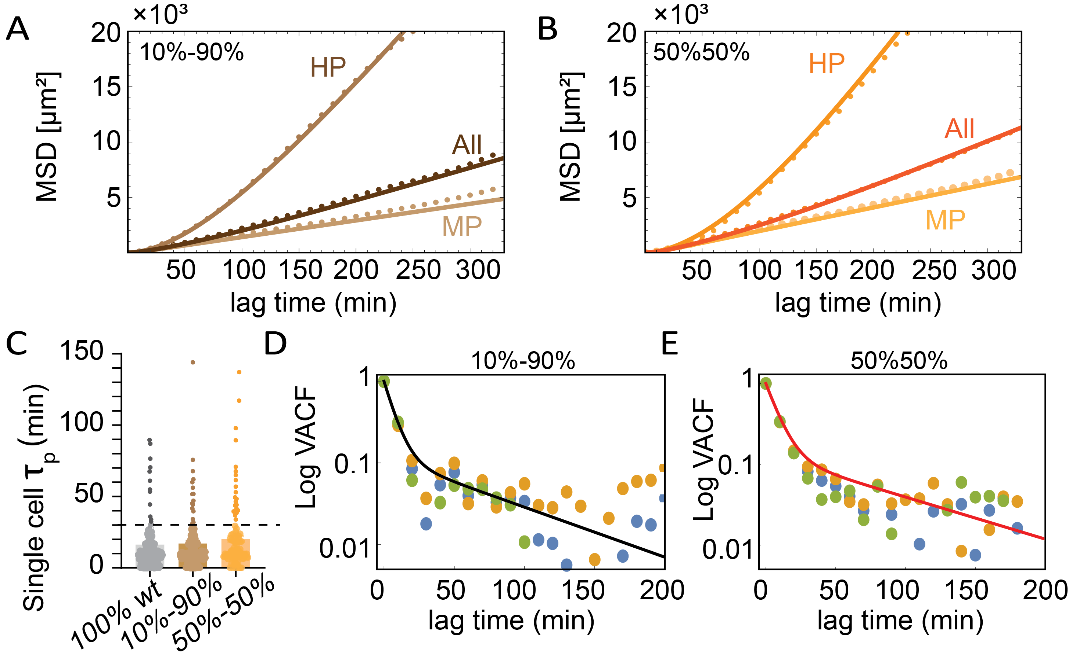
**

**Figure S8: EGFRvIII cells sustain and enrich the highly persistent EGFRwt cell subpopulation, exhibiting improved motility patterns and a rise in percentage. A-B**: Mean squared displacement plots for each EGFRwt cell- subpopulation (MP, HP) and all cells in each condition; 10%vIII (**A**) and 50%vIII (**B**). The dotted lines represent the experimental MSD values and the lines represent the theoretical-based MSDs (R^2^>0.93). **C**: Quantification of single-cell time persistence ($\tau$) along the primary axe of EGFRwt cells in control (brown) and dasatinib (gray). **D-E**: Log of Velocity Autocorrelation Function (VACF) decays over time from separate VACF fits (R^2^>0.95) of each EGFRwt cell subpopulation (MP- and HP- τ).

**
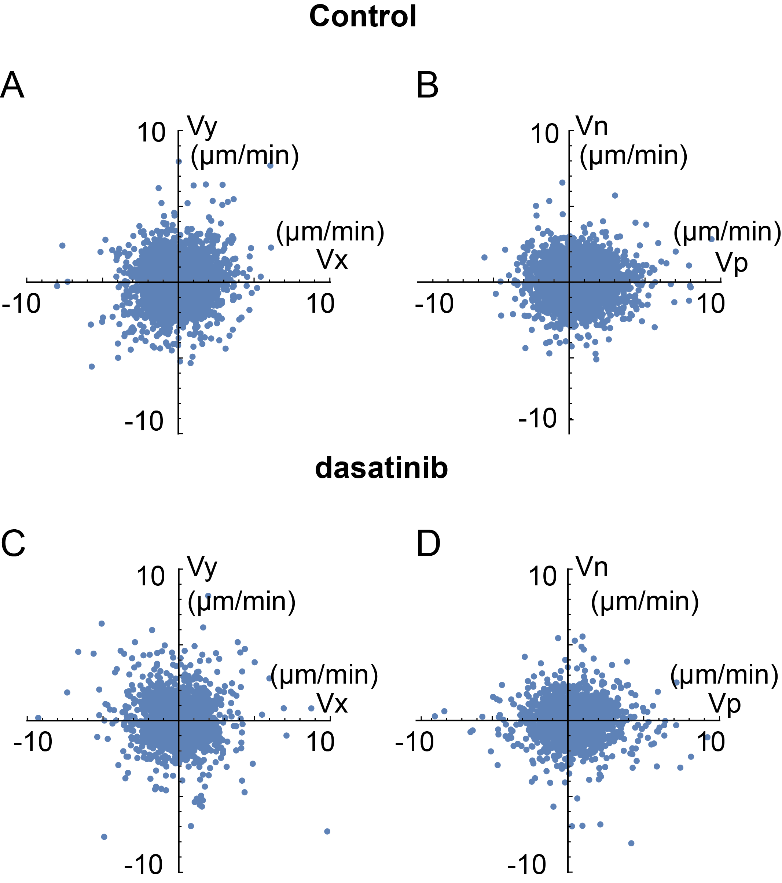
**

**Figure S9: Src inhibition does not affect cells’ anisotropic migration. A-D**: Scatter plots showing the instantaneous velocity components of Control (**A, B**) and dasatinib treated cells (**C, D**). The left plots display (A, C) velocities’ distribution in x and y axis and the right plots (B, D) show this distribution in the primary (V_p_) and non-primary axis of migration (V_n_).

**References**

1. Czirók, A., Schlett, K., Madarász, E., and Vicsek, T. (1998). Exponential distribution of locomotion activity in cell cultures. Phys. Rev. Lett. https://doi.org/10.1103/PhysRevLett.81.3038.

2. Selmeczi, D., Mosler, S., Hagedorn, P.H., Larsen, N.B., and Flyvbjerg, H. (2005). Cell motility as persistent random motion: Theories from experiments. Biophys. J. https://doi.org/10.1529/biophysj.105.061150.

3. Wu, P.H., Giri, A., Sun, S.X., and Wirtz, D. (2014). Three-dimensional cell migration does not follow a random walk. Proc. Natl. Acad. Sci. U. S. A. https://doi.org/10.1073/pnas.1318967111.

4. Nousi, A., Søgaard, M.T., Audoin, M., and Jauffred, L. (2021). Single-cell tracking reveals super-spreading brain cancer cells with high persistence. Biochem. Biophys. Reports. https://doi.org/10.1016/j.bbrep.2021.101120.

5. Stokes, C.L., Lauffenburger, D.A., and Williams, S.K. (1991). Migration of individual microvessel endothelial cells: Stochastic model and parameter measurement. J. Cell Sci. https://doi.org/10.1242/jcs.99.2.419.

6. Dickinson, R.B., and Tranquillo, R.T. (1993). Optimal estimation of cell movement indices from the statistical analysis of cell tracking data. AIChE J. https://doi.org/10.1002/aic.690391210.
